# Computer simulation of human leukocyte antigen genes supports two main routes of colonization by human populations in East Asia

**DOI:** 10.1186/s12862-015-0512-0

**Published:** 2015-11-04

**Authors:** Da Di, Alicia Sanchez-Mazas, Mathias Currat

**Affiliations:** Department of Genetics and Evolution - Anthropology Unit, Laboratory of Anthropology, Genetics and Peopling history (AGP lab), University of Geneva, 12 rue Gustave-Revilliod, Geneva, CH-1211 Geneva 4 Switzerland; Institute of Genetics and Genomics in Geneva (IGE3), University of Geneva Medical Centre (CMU), 1 rue Michel-Servet, Geneva, CH-1211 Geneva 4 Switzerland

**Keywords:** East Asia, Human peopling history, HLA, Computer simulation, Approximate Bayesian computation, Balancing selection

## Abstract

**Background:**

Recent genetic studies have suggested that the colonization of East Asia by modern humans was more complex than a single origin from the South, and that a genetic contribution via a Northern route was probably quite substantial.

**Results:**

Here we use a spatially-explicit computer simulation approach to investigate the human migration hypotheses of this region based on one-route or two-route models. We test the likelihood of each scenario by using Human Leukocyte Antigen (HLA) − A, −B, and − DRB1 genetic data of East Asian populations, with both selective and demographic parameters considered. The posterior distribution of each parameter is estimated by an Approximate Bayesian Computation (ABC) approach.

**Conclusions:**

Our results strongly support a model with two main routes of colonization of East Asia on both sides of the Himalayas, with distinct demographic histories in Northern and Southern populations, characterized by more isolation in the South. In East Asia, gene flow between populations originating from the two routes probably existed until a remote prehistoric period, explaining the continuous pattern of genetic variation currently observed along the latitude. A significant although dissimilar level of balancing selection acting on the three HLA loci is detected, but its effect on the local genetic patterns appears to be minor compared to those of past demographic events.

**Electronic supplementary material:**

The online version of this article (doi:10.1186/s12862-015-0512-0) contains supplementary material, which is available to authorized users.

## Background

During their extensive colonization of the world since the emergence of *Homo sapiens* in East Africa around 200,000 years ago [1], modern human populations have evolved genetically through various mechanisms including random genetic drift, gene flow and natural selection [2]. While the effects of genetic drift and gene flow on the genetic variation within and among different populations are strongly related to population demography, those of natural selection depend on environmental pressures and may blur the signals of population demography. To reconstruct the migration history of anatomically modern humans throughout the world, an essential issue is thus to estimate the respective contribution of these different evolutionary forces, in particular when the studied genes are known to evolve under substantial natural selection.

A topic which has been heavily debated over the last few years is the human peopling history of East Asia, mostly because Northern East Asian populations (NEAs), on one side, and Southern East Asian populations (SEAs), on the other side, have been found to differ greatly from each other from a genetic point of view [3]. Indeed, while population geneticists generally accept the idea of a recent African origin of East Asian populations (less than 100,000 years ago) despite possible, but minor contributions from other human species once inhabiting Eurasia, Neanderthals and/or Denisovans [4] for instance, two main alternative scenarios have been proposed to explain the first arrival of modern humans in this region. According to the Southern-origin model, modern humans migrated eastward along the southern edge of the Himalayan Mountains to reach the South of East Asia (SEA), and further differentiated by expanding northward to the North of East Asia (NEA) [5–7]. Some authors defending the Southern-origin hypothesis also admit a certain amount of gene flow from Central Asia at a recent period beginning 3,000 or 2,000 years ago [3]. An alternative scenario is known as the Pincer model [8]. It suggests that, besides the aforementioned Southern route, modern humans also followed a Northern route across Central Asia (along the northern edge of the Himalayas) and Southern Siberia to reach NEA [9].

**Fig. 1:**
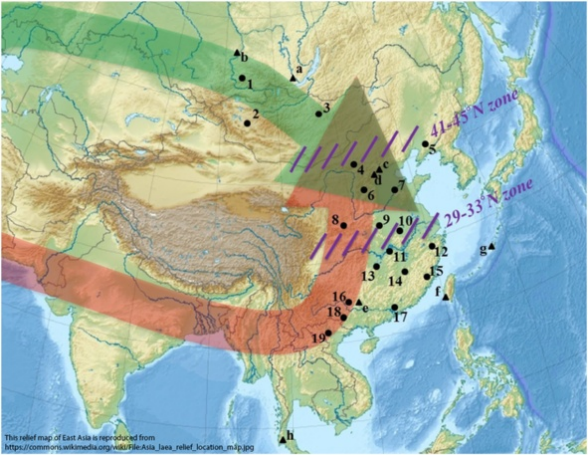
Topographic map of East Asia showing hypothesized modern human migration routes and suggested barriers. Northern and Southern routes are represented by green and red arrows, respectively, while the two contact zones are marked in purple. Triangles indicate representative Upper Paleolithic archeological sites with human remains: a. Mal’ta; b. Afontova Gora-2; c. Upper Cave; d. Tianyuan Cave; e. Liujiang; f. Chochen; g. Minatogawa; h. Niah Cave and points indicate modern populations samples for HLA−A, −B, −DRB1 loci: 1. Tuvinians; 2. Oold; 3. Khalkha; 4. Mongolians; 5. Liaoning Han; 6. Shanxi Han; 7. Shandong Han; 8. Xi’an Han; 9. Henan Han; 10. Anhui Han; 11. Hubei Han; 12. Zhejiang Han; 13. Hunan Han; 14. Jiangxi Han; 15. Fujian Han; 16. Maonan; 17. Guangdong Han; 18. Zhuang; 19. Muong (see Additional file 1: Table S1 and Additional file 2: Table S2)

Interestingly, the sharp genetic variation between NEAs and SEAs has not solely been observed for non-functional, supposedly neutral, genetic markers such as Short Tandem Repeats (STRs) and Single Nucleotide Polymorphisms (SNPs). Indeed, similar patterns have been found for the immune-system genes defining the human leukocyte antigen (HLA) polymorphisms [10–12], the evolution of which is partly driven by heterozygous advantage in pathogen-rich environments [13–15]. To account for the specific observations made for this system in East Asia (i.e. a continuous genetic differentiation along the latitude shaped by two groups of lineages and alleles showing clinal distributions, and a significant decrease of genetic diversity from north to south), a third scenario, named the Overlapping model, has recently been proposed (Fig. 1). This scenario is basically similar to the Pincer model suggesting two routes of colonization into East Asia, the difference being that it emphasizes the role of large-scale and probably long-lasting genetic exchanges between NEAs and SEAs in a northern geographic region, which eventually created the observed north-south genetic continuity [10–12].

HLA genes provide an interesting complement to neutral variability for the study of human peopling history because they may reflect additional selective processes beyond the effect of past demography and migration. Their results may thus be contrasted to those obtained with neutral loci. HLA genes constitute very useful data to tackle the question of the settlement history of East Asia because allelic states (in the form of HLA lineages defined at the 1^st^-field level of resolution according to the official nomenclature found at http://hla.alleles.org/nomenclature/naming.html) are available at several loci for the same sets of individuals in a large database of East Asian populations [10–12], thus avoiding the difficulty to consider sampling variation or heterogeneity among loci [16]. To our knowledge, there are currently no other genetic datasets available for East Asian populations which are equivalent in terms of both sample numbers and sample sizes. A potential difficulty, however, is that HLA genes partly evolve under balancing selection, which may create confounding effects. Indeed, different evolutionary forces, which are hard to disentangle by classical population genetics approaches, sometimes generate similar signals on patterns of genetic variation. For instance, genetic diversity within populations may be maintained through intensive gene flow but also through balancing selection; on the contrary, a loss of diversity may be the result of either rapid genetic drift or purifying selection [17]. Moreover, a continuous pattern of genetic variation among populations (often described by genetic clines) can be explained by demographic processes (e.g. demic diffusion with admixture between genetically distinct populations [18], isolation-by-distance with gene flow between neighboring populations [19, 20], population expansions [21]) or varying selective pressures in different environments [22]. However, such confounding effects are not expected to affect all HLA loci in the same way. Indeed, previous studies have shown that, whereas HLA–B and –DRB1 exhibit significant excess of heterozygotes in most populations tested so far, suggesting balancing selection, the genetic diversity observed at HLA−A is closer to neutral expectations [23, 24], and is thus more susceptible to reveal demographic signals. Therefore, we decided to tackle the problem mentioned above both by undertaking an original computer-simulation analysis allowing to disentangle the effects of distinct evolutionary forces and by applying this approach to three different HLA loci known to be submitted to distinct intensities of selection, namely HLA–A, –B and –DRB1, which were also tested on identical population samples available in our large database for East Asian populations. Our original computer-simulation algorithm allows a direct comparison between the three HLA genes investigated here in controlling for demography, hence a main interest of our study. When combined with Approximate Bayesian Computation (ABC) [25], it allows to estimate separately the influence of demography and the strength of selection on each locus, even in the absence of additional “control” neutral datasets. This approach has already proved its effectiveness in the field of population genetics, e.g. [16, 26, 27]. In this study, we have specifically adapted it to reproduce East Asian HLA–A, –B and –DRB1 genetic variation under the three hypothetical models described above taking into account various evolutionary parameters including population density, demographic growth, migration, and balancing selection.

## Methods

### Simulations

**Fig. 2:**
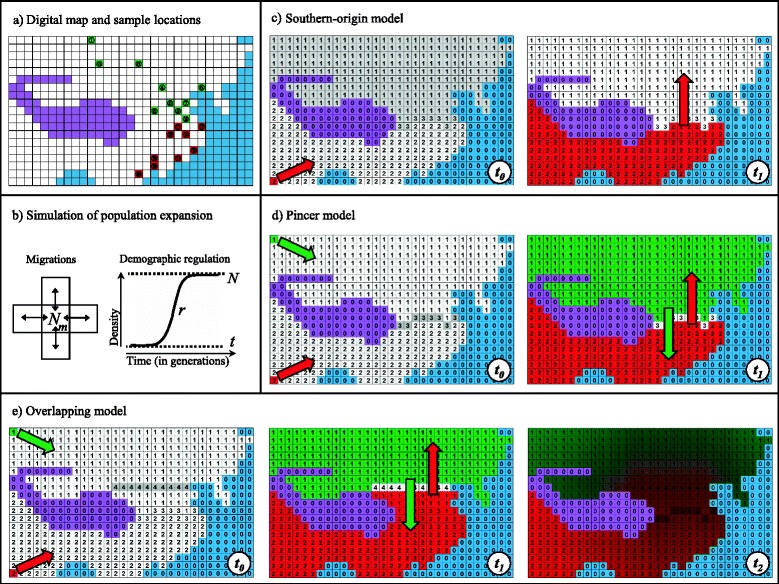
Schematic representation of simulation of population expansion and demographic models. **a** location of sampled populations on the digital map, population numbers corresponding to those in Fig. 1 and Additional file 1: Table S1; (**b**) population dynamics in one deme; (**c**-**e**) the three models we simulated (number in each deme of represents its corresponding zone number: zone-0: uninhabitable area; zone-1: North of East Asia; zone-2: South of East Asia; zone-3: 29-33°N ; zone-4: 41-45°N)

A simulation program called SELECTOR was developed by our laboratory in recent years, written in C^++^ and compiled on Linux environment. The main algorithms of the program have been described and applied in a previous study focusing on human genetic differentiations across the Strait of Gibraltar [16]. The program simulates populations of diploid individuals from generation to generation, forward in time, within a “stepping-stone” framework [28], using a set of predefined parameters with prior distributions. The geographical region of interest is represented by a digital map subdivided into demes with equal size of 40,000 (200 × 200) km^2^ (Fig. 2a). Generations are discrete, which means that individuals from one generation, in each deme, are completely replaced by their descendants in the next generation. The population density (*N*) increases logistically (with growth rate *r*) until reaching the maximum carrying capacity of the deme (Fig. 2b). A proportion of individuals belonging to each deme (the migration rate *m*) migrates to neighboring demes (spread equally among neighbors, Fig. 2b), allowing a population expansion into all demes from one or two source demes. The genotype of each diploid individual (homozygote or heterozygote) is composed by lineages and is created randomly from a common ancestral pool of lineages (Number of initial lineages *A*) at the first generation, then from the parental pool of the same deme for all subsequent generations.

Geographic barriers to ancient human migrations were defined as uninhabitable demes, either permanently, like seas and Himalayan Mountains, or temporarily, like great rivers, climate limits, and/or cultural frontiers (Fig. 2). SELECTOR was adapted to the current study by defining two different zones, NEA and SEA (see next section), accounting for the well documented north-south genetic variation in East Asia. NEA and SEA are separated by the 29-33°N zone, which represents the area of Qinling-Huaihe line or Yangtze River.

In each deme, SELECTOR simulates the evolution of lineage frequencies of a gene by considering as simulation parameters various demographic factors (*N*, *m*, *r*). In addition, SELECTOR may introduce the effect of balancing selection on a gene by giving advantage (depending on a selection coefficient *s*) to heterozygotes compared to homozygous individuals when creating a new generation, which corresponds to the Symmetric Overdominant Selection model (SOS [16]). For evaluating the robustness of our results regarding the choice of the selection model, we tested two alternative balancing selection models with a relatively smaller number of simulations: heterozygote advantage varying along latitude (LOS for Latitudinal Overdominant Selection) and Negative Frequency-Dependent Selection (NFDS). The total number of generations corresponds to about 60,000-50,000 years of the overall modern human peopling history in East Asia, according to archaeological and genetic studies [5]. The modified version of SELECTOR used here allows division of the total simulation duration into several periods with different demographic characteristics. It allows to take into consideration critical scenarios such as the emergence or disappearance of geographic barriers (by changing *m*), or the Paleolithic-Neolithic transition (by changing *N and r*). At the end of a simulation, SELECTOR samples individuals in each cell representing a geographic location where real genetic samples are available, with sample sizes being identical to real data (Fig. 2a). This approach allows a direct comparison between simulated and observed data.

Different mechanisms such as point mutations, recombinations and gene conversions frequently introduce new HLA variants in human populations [29, 30], the generation of new alleles (i.e. defined at 2^nd^-field level of resolution or above, according to the official nomenclature) being thus difficult to model. In contrast, HLA lineages are more conservative and considered to be ancient [31, 32]. As we do not use HLA allelic but lineage data in this study (both to take advantage of the large amount of lineage data available at several HLA loci for the same population datasets and to avoid the challenging issue of modelling complex mechanisms for the generation of new HLA alleles), SELECTOR does not simulate the appearance of new alleles, but simulates changes in lineage frequencies due to genetic drift and possible balancing selection.

### Demographic models

Because complex models often lead to biased estimations when using ABC, it is recommended to simulate a very large set of data under a simplified model with a restricted number of parameters [33]. For each of the three scenarios that we have simulated, we tested several alternative versions in a qualitative way in order to find the most representative but simplified models presented below, and we performed 100,000 simulations according to each final scenario for further quantitative estimations. NEA and SEA were always defined as zone-1 and zone-2.

1. Southern-origin model (one-route model, Fig. 2c). One expansion initiates from the southwestern-most deme at generation *t*_*0*_ *=* 0 (between 60,000 and 50,000 years ago), the duration of a simulation being 2,500 generations. A complete barrier to migration corresponding to the 29-33°N zone (zone-3) exists, which prevents the passage of migrants. At generation *t*_*1*_ (0 < *t*_*1*_ < 2,000), the barrier either disappears or becomes a partial barrier to gene flow according to the specific parameters of simulation. Maximum *t*_*1*_ was set to 2,000 generations to allow the NEA to be entirely colonized at the end of the 2,500 generations.
2. Pincer model (two-route model, Fig. 2d). Two expansions initiate from the southwestern-most and northwestern-most demes, respectively, at generation *t*_*0*_ = 0 (between 60,000 and 50,000 years ago). Individuals in these two demes are derived from a common ancestral source population. Like for the Southern-origin model, a complete barrier to migration corresponding to the 29-33°N zone (zone-3) exists until its disappearance, at least partially, at generation *t*_*1*_ (0 < *t*_*1*_ < 2,500).
3. Overlapping model (two-route model, Fig. 2e). Two expansions initiate from the southeastern-most and the northeastern-most demes, respectively, at generation *t*_*0*_ = 0 (between 60,000 and 50,000 years ago). A complete barrier corresponding to the 41-45°N zone (zone-4) exists until its disappearance, at least partially, at generation *t*_*1*_ (0 < *t*_*1*_ < 2,500). This barrier to gene flow is located further north than the 29-33°N zone (zone-3). The latter also constitutes a partial barrier emerging later and reducing gene flow considerably with migration rate of 0.1 *m* at generation *t*_*2*_ (2,500 < *t*_*2*_ < 3,000).

In summary, the three models have 12 parameters in common: number of initial lineages in the source population *A*, selection coefficient *s*, north-south separation time *t*_*1*_, as well as independent *N*, *m* and *r* for the NEA, the SEA and the barrier zone. For the two-route models, we were particularly interested by the admixture time *T* (*T* = 2,500-*t*_*1*_ for the Pincer models and *T* = *t*_*2*_-*t*_*1*_ for the Overlapping model) between NEAs and SEAs. Note that the Southern-origin and Pincer models run for 2,500 generation in total while the Overlapping model runs for 3,000 generations. The product of *N* and *m* (*Nm*) was estimated as a whole because it represents the absolute number of migrants leaving a deme. The prior distributions of parameters were defined based on literature (Table 1).

**Table 1:** Prior distributions and description of the parameters used in the simulations

Parameters	Meaning	Prior distribution	References and/or explanation
*T*	Admixture time between NEA and SEA under two-route models (in generations)	1 to 2,500	Between 0 and 65,000 years (estimation of the spread of modern humans in East Asia [5]), each generation representing 25 years
*A*	Number of initial lineages in the source population	10 to 50	Numbers of HLA−A, −B and −DRB1 lineages vary between 10 and 40 in East Asian samples (from data listed in Additional file 1: Table S1)
*N*	Maximum population density of one deme	0 to 5,000	According to the estimation of hunter-gatherer densities at the end of Paleolithic (0-0.4 individuals per *km* ^2^ [18]
*m*	Population migration rate of one deme	0.01 to 0.20	Between 1 % and 20 % of emigration per generation [18]
*r*	Population growth rate of one deme	0.01 to 0.20	According to the estimation of growth rate for Paleolithic populations [18]
*s*	Coefficient of balancing selection	0 to 0.025	Selection rate higher than 2.5 % was tested by preliminary simulations but it never lead to the reproduction of observed data [40–42]

### Observed data

The major objective of the simulation approach is to reproduce the general genetic pattern of East Asian populations and to estimate parameters at a large geographic scale. In order to avoid potential bias due to local demographic events specific to certain populations, such as bottlenecks, directional selection or recent gene flow, we excluded some populations known for their small size and/or isolation, such as the Nu, Wa, Jinuo [34], or for their recent interbreeding history, such as the Uyghurs, Kinh and Thai [35]. We also excluded populations from peninsula and island areas, such as Koreans, Taiwanese aborigines, Japanese and Ryukyuans because these populations may have been subject to strong founder effect or admixed origins which are more difficult to model in our study [35–38].

In total, 21 lineages were considered for HLA−A (A*01, *02, *03, *23, *24, *25, *26, *34, *66, *11, *29, *30, *31, *32, *33, *74, *68, *69, *36, *43, *80), 34 for HLA−B (B*07, *08, *13, *14, *15, *18, *27, *35, *37, *38, *39, *40, *41, *42, *44, *45, *46, *47, *48, *49, *50, *51, *52, *53, *54, *55, *56, *57, *58, *59, *67, *73, *78, *81) and 14 for HLA−DRB1 (DRB1*01, *02, *03, *04, *07, *08, *09, *10, *11, *12, *13, *14, *15, *16). Due to the relatively abundant population data tested for these HLA lineages, we managed to keep 19 populations (average sample size 2,662, ranging from 52 to 9,678 individuals), each of them being typed on the same samples for the three loci in the same study (Figs. 1, 2a and Additional file 1: Table S1). This enabled us to make a direct comparison among the loci by excluding possible sampling bias for the same population. All HLA frequency data used in this study were taken from publicly available publications (see references in Additional file 1: Table S1).

### Model comparison

A total of 12 summary statistics were carefully chosen to capture a maximum of information from the simulated and observed data, at various levels of genetic diversity (locus variability, intra-population diversity overall but also separately in NEAs and SEAs, gradient of diversity along latitude, inter-population differentiation overall, between and within NEAs and SEAs groups, Table 2). The statistics were estimated by arlsumstat program [39]. The ABCtoolbox package was used to perform model comparisons and later parameter estimations [33]. The likelihood of a model was evaluated by computing Euclidean distances between the simulated summary statistics and those of the observed data [33, 40, 41]. To evaluate whether the observed data were in agreement with the simulated data, ABCtoolbox reports a *p-value* varying between 0 (no fit) and 1 (good fit) [33].

**Table 2:** Statistics computed from observed data for the three HLA loci

Statistic	Meaning	A	B	DRB1
*M*_*A*_	Mean of lineage number for all populations	14.47	27.26	12.84
*SD*_*A*_	Standard deviation of lineage number for all populations	3.96	6.64	1.01
*M*_*H*_	Average of heterozygosity index *H* for all populations	0.79	0.91	0.89
*SD*_*H*_	Standard deviation of *H* for all populations	0.054	0.021	0.019
*M*_*H-North*_	Mean of *H* for NEAs	0.84	0.92	0.90
*SD*_*H-North*_	Standard deviation of *H* for NEAs	0.012	0.0056	0.0070
*M*_*H-South*_	Mean of *H* for SEAs	0.75	0.89	0.88
*SD*_*H-South*_	Standard deviation of *H* for SEAs	0.044	0.010	0.021
*R*_*H-lat*_	Correlation coefficient between *H* and latitude	0.78	0.84	0.65
*F*_*ST*_	*F* statistic measuring within- and inter-group differentiation	0.017	0.011	0.0075
*F*_*CT*_	*F* statistic measuring inter-group differentiation	0.015	0.0092	0.0055
*F*_*SC*_	*F* statistic measuring within-group differentiation	0.0023	0.0016	0.0020

The relative probabilities of the three scenarios were computed using the model choice acceptance method [42] consisting in the proportion *δ* of data sets, among the *N* simulated, that are the closest to the observed data set. Here, *N* is equal to 300,000 simulations (100,000 per scenario), and we used three different values of *δ* to ensure the robustness of the comparison: *δ* = 0.25 % (750 simulations), 0.5 % (1,500 simulations) and 1 % (3,000 simulations). In addition, ABCtoolbox allows performing model selection through the computation of the Bayes factors. The Bayes factor *B*_*AB*_ in favor of model *M*_*A*_ over model *M*_*B*_ is *B*_*AB*_ = *fM*_*A*_*(s*_*obs*_*)/fM*_*B*_(*s*_*obs*_) where *fM*_*A*_ and *fM*_*B*_ are the marginal densities of model A and B, respectively.

### Estimation of parameters and validation

In order to optimize the amount of information on the model parameters extracted from the data, we further calculated Partial Least Squares (PLS) on the original statistics [43], a method available in ABCtoolbox [33] and similar to the Principal Component Analysis in extracting the main independent axes of variance. A Root Mean Squared Error Prediction (RMSEP) chart is reported for each parameter, reflecting how its value influences the simulated results and allowing to decide the best number of PLS to use for parameter estimation.

We estimated the parameters under the most likely model using the ABC-GLM method implemented in ABCtoolbox [44]. This method initiates a process of rejection by retaining a small proportion of simulations based on the minimized Euclidean distance calculated between the simulated and observed statistics (with or without PLS). The posterior distribution for each parameter is computed from these retained simulations [33]. We set the proportion of retained simulations as 0.5 % but also tested other fractions (0.25 %, 1 %) to ensure the robustness of the estimation.

The results obtained were validated by studying the distribution of “posterior quantiles” for each parameter, in order to test whether the posterior distribution of the parameters is biased compared to the prior distribution. A total of 100 sets of statistics were generated under the best model with parameters drawn from the posterior distributions and considered as if they had been observed in reality (“pseudo-observations”). It has been proved mathematically [45] that the positions of these true parameters are distributed uniformly in the marginal cumulative posterior distribution. Deviation from the uniform distribution was detected by the Kolmogorov-Smirnov test implemented in ABCtoolbox [33]. Moreover, the simulated and retained values for each pair of statistics were plotted to verify whether the observed values fell within the range of the retained ones (Additional file 3: Table S3).

### Computation platform

Our computation tasks were sent to a platform called EZ-Grid composed of hundreds of nodes in several locations in Switzerland and France [46].

## Results

### Likelihood of the different models tested

**Fig. 3:**
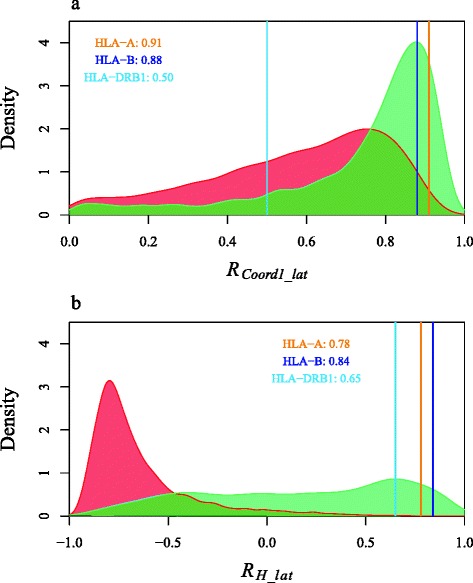
Density distribution of simulated correlation coefficient between latitude and among- or within-population variation indices. Density distribution of: (**a**) absolute value of correlation coefficient between the first Principal Coordinate and latitude of populations and (**b**) value of correlation coefficient between heterozygosity index *H* and latitude of populations for one-route model and two-route model, respectively (red curve: one-route model; green curve: two-route model; observed values for each HLA locus are listed and displayed by vertical lines: orange line: HLA−A, blue line: HLA−B, light blue line: HLA−DRB1)

At among-population level, the north-south differentiation is more frequently observed under the two-route models, with higher values of correlation coefficient between the first coordinate of Principal Coordinate Analysis and latitude of populations (|*R*_*coord1-lat*_|, Fig. 3a). At within-population level, the simulated genetic diversity (*H*) decreases systematically from south to north under the Southern-origin model, leading to negative values of *R*_*H-lat*_ in the majority (>95 %) of simulations (Fig. 3b). Under the two-route models, *R*_*H-lat*_ values thus vary between -1 and +1 (Fig. 3b), depending on several parameters including the product of population density by migration rate *Nm* and the selection coefficient *s*.

When the results of the 300,000 simulations are mixed, the proportion found for each model among the 1,500 best simulations retained (*δ* = 0.5 %) indicates that the Pincer and the Overlapping models are much more likely (>96 % combined, Table 3) than the Southern-origin model, the latter being almost not supported (<4 %). Similar results are obtained with either 750 (*δ* = 0.25 %) or 3,000 (*δ* = 1 %) best simulations retained.

**Table 3:** Model comparison using retained simulations. Proportions of simulations (%) under each of the three models among 750, 1,500 and 3,000 best simulations retained from 300,000 simulations (100,000 for each model)

Number of retained simulations	Locus	Southern-origin model	Pincer model	Overlapping model
750	A	2.4	31.2	66.4
	B	0.5	26.3	73.2
	DRB1	0.2	37.5	62.3
1,500	A	3.8	33.1	63.1
	B	0.7	27.3	71.9
	DRB1	0.3	48.1	51.6
3,000	A	5.4	47.0	47.6
	B	1.4	40.4	58.2
	DRB1	1.0	48.8	50.2

In addition, the Bayes factors are extremely in favor of the Pincer and Overlapping (both two-route) models because of the low marginal density computed for the Southern-origin model (Table 4). The Bayes factor computed between models also “decisively” [47] supports better the Overlapping than the Pincer model (between 5,000 and 40,000 times depending on the locus, Table 4).

**Table 4:** Model comparison using marginal density and Bayes’ factor. Marginal density and *p-values* were output by ABCtoolbox for each of the three models, while the Bayes’ factors were computed between each pair of models. (3,000 best simulations retained (1 %) from 300,000 simulations, 100,000 for each model)

Model	Locus	HLA−A	HLA−B	HLA−DRB1
Southern-origin model	*p-value*	0	0	0
	*Marginal density*	3.58E-27	1.93E-90	198
Pincer model	*p-value*	0.01	0.05	0.08
	*Marginal density*	0.79	1.17E4	1.22E6
Overlapping model	*p-value*	0.07	0.08	0.24
	*Marginal density*	8.20E3	6.00E7	4.99E10
Bayes’ factor in favor of Pincer model to the Southern-origin model		2.20E26	6.06E93	6.16E3
Bayes’ factor in favor of Overlapping model to the Southern-origin model		2.29E30	3.12E97	2.52E8
Bayes’ factor in favor of Overlapping model to the Pincer model		1.04E4	5.12E3	4.09E4

The simulations of the Southern-origin model are never able to reproduce the observed HLA−A, −B and −DRB1 data, as indicated by the *p-values*, equal to 0 for the three loci. For both two-route models, the *p-values* are higher, reaching 1-8 % for the Pincer model and of 7-24 % for the Overlapping model (Table 4). Plots of simulated, retained and observed data (Additional file 3: Table S3) for each pair of statistics further show the goodness of the Overlapping model to reproduce simultaneously different statistics, and the model fits better the HLA−B and −DRB1 loci than the HLA−A locus. In conclusion, the results allow us to reject the Southern-origin model as a relevant hypothesis to explain the East Asian HLA genetic variation. They also “decisively” [47] favor the Overlapping model over the Pincer model.

Both alternative models of selection (LOS and NFDS) give similar results for scenario comparison (Additional file 4: Table S4 and Additional file 5: Table S5). For all loci, the Overlapping scenario is the most likely (>52 % relative probability under LOS and 38-85 % under NFDS, respectively) and the Southern route scenario significantly the less likely (<1 % under both LOS and NFDS). The only exception is HLA−A under NFDS which reaches a support up to 28 % while still being the worst scenario. The Pincer model is always intermediate, with a relative probability between 30-47 % under LOS and 14-44 % under NFDS, respectively.

### Posterior distribution of parameters

For the most likely scenario (Overlapping) we have computed the Bayes factors between the selection models SOS and LOS and we found a decisive support to SOS over LOS for all loci (Bayes factors > 2,700). Consequently, we estimated the parameters under the Overlapping scenario and SOS selection model. According to the RMSEP charts (Additional file 6: Table S6), two locus-related parameters, i.e. the initial number of lineages (*A*) and the selection coefficient (*s*), affect most statistics under the Overlapping model. Regarding demography, the duration of admixture (*T*) appears as an essential factor, and the product of population density by migration rate (*Nm*) also has considerable influence. When *N* and *m* are treated separately, we notice that *m*, rather than *N*, is the decisive factor. By contrast, the growth rate (*r*) has very slight influence on the results, so do the other parameters. The posterior distribution of these parameters was estimated with 6 PLS under the Overlapping model, which resulted in relatively high *p-values* (>29 %).

**Fig. 4:**
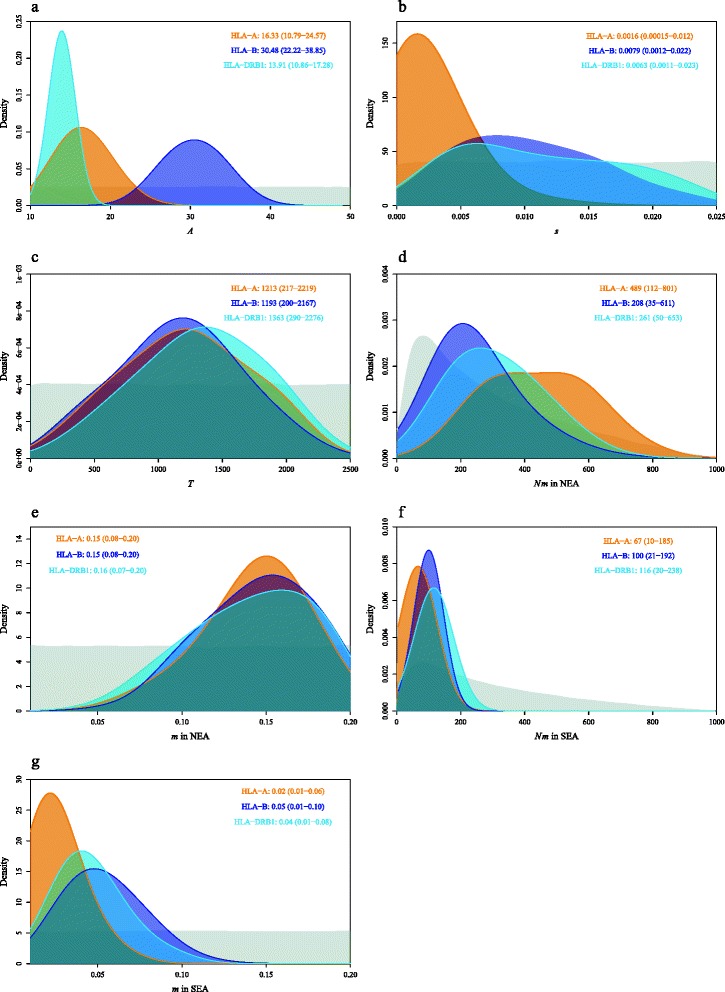
Posterior distribution of parameters estimated with 6 PLS under the Overlapping model for HLA−A, −B, −DRB1 loci. Mode of the distribution and 95 % HPD Interval are given for (**a**) allele number *A*, (**b**) selection rate *s*, (**c**) admixture time *T*, as well as (**d**,**f**) product of population density and migration rate *Nm* and (**e**,**g**) migration rate *m* in NEAs and in SEAs (orange curve: HLA−A, blue curve: HLA−B, light blue curve: HLA−DRB1; grey curve: the prior distribution)

The graphical representations of 95 % posterior distributions of the estimated parameters of interest are reported in Fig. 4. The estimation of the demographic parameters is consistent among the different loci: *Nm* in NEA is considerably higher compared to *Nm* in SEA (Fig. 4d, f), while the most probable *T* values lie between 1,193 and 1,363 generations, representing at least 24,000 years (Fig. 4c). By contrast, the estimated values of *A* and *s* clearly differ among the loci. While *A* is related to the number of lineages of the corresponding locus (Fig. 4a), *s* reveals that balancing selection shaped the three HLA loci differently, the effect of which is greater for HLA−B and HLA−DRB1 compared to HLA−A (Fig. 4b).

### Validation of results

The validation procedure using the Kolmogorov-Smirnov test (Additional file 7: Table S7) confirmed that the prior distribution was not biased for any parameter except the number of lineages *A*, the latter revealing a much larger prior distribution compared to the posterior distribution, because an identical prior distribution was defined for the three different HLA loci (Table 1). Plots of simulated, retained and observed data for each pair of statistics further show a good coverage of posterior distribution by the prior distribution of parameters, which is true for each locus (Additional file 3: Table S3).

## Discussion

### Effectiveness of our simulation approach and reliability of results

The peopling scenarios we simulated have been designed to capture the main processes as simply as possible in order to limit considerably the parameters and thus increase the likelihood of ABC estimation. The prior distributions of parameters were carefully designed in order to create a set of non-biased simulated data. Comparison of models has been performed both by the model choice acceptance method and by using Bayes factors between each pair of models, two different ways leading to identical conclusions. One advantage of the Bayesian approach used here (ABC) is that models with different numbers of parameters can be directly compared thanks to the prior distributions. Finally, the parameters were estimated under the most likely model, which has been shown to be reliable according to independent validation procedures.

### A significant contribution of the Northern route

Our results show that the Southern-origin model can be rejected as it is almost never able to reproduce the observed data in East Asia, whatever the initial conditions are. A formal comparison of the three models using the ABC approach indicates that both two-route models (Pincer and Overlapping) are significantly more likely than the Southern-origin model (>96 % among 1,500 retained simulations) for the three HLA loci (Table 3). The result of this direct comparison is confirmed by the “decisive” [47] Bayes factor (>6,000 for all loci, Table 4). These simulation results thus very strongly support the crucial role of the Northern route in the peopling history of East Asia: human expansion(s) through a unique Southern route is not sufficient to explain the observed HLA genetic patterns.

When comparing our results to those obtained with putatively neutral variability, we find that, interestingly, a recent study on abundant Y chromosome data from China reached a similar conclusion to ours, supporting a main Southern route of migration for the peopling of East Asia but also an ancient Northern route, following the LGM period about 18,000 years ago, for the ancestors of current East Asian males [48]. In addition, a recent paper by Zhao et al. [49] combining new mtDNA data from 89 to 3,000 years old human remains of northern China with other published genetic data showed that the genetic structure of East Asian populations into a northern and a southern group was already shaped 3,000 years ago, suggesting a substantial mtDNA genetic contribution of Northern East Asian populations to the East Asian genetic pool in the past. Another study investigating genome-wide SNP diversity in Central and East-Asia [50] found (like us) that haplotype diversity is strongly correlated with latitude as a result of prehistoric population divergence, but suggested that the geographic sources contributing to East Asian populations were mainly from South East Asia with a minor contribution from Central Asia. Nevertheless, the dataset used by these authors is hardly comparable to ours because it is based on the analysis of 10 “combined” populations, among which only 4 correspond to Chinese groups and North-East Asian are largely underrepresented, as emphasized in a former paper [22]. Therefore, our results tend to reflect those of related studies carried out on putatively neutral markers by supporting a two-route model of migration in East Asia, with an emphasis on the role of the second route north to the Himalaya. This suggests that the influence of demography on HLA genes is stronger than selection when studying evolutionary processes at this geographical and temporal scales, as further discussed below.

Further comparisons between the two two-route models indicate a greater likelihood of the Overlapping model compared to the Pincer model, according both to the acceptance method (Table 3) and to the Bayes factor (>5,000 thus “decisive” [47], Table 4). This is in agreement with the previous conclusions based on a detailed analysis of HLA lineage and allele frequency variation [10] and on archaeological data [51, 52]. In addition, under the Overlapping model, the *p-values* computed on the raw statistics are always above 5 %, which is not the case under the Pincer model (1-8 %).

These results are robust to the choice of balancing selection model, thus accounting for the uncertainty of the mode of selection on HLA [53]. Indeed, a model of Symmetric Overdominant Selection varying in space (LOS) or Negative Frequency-Dependent Selection (NFDS) gives similar scenario comparisons to those obtained with the model of uniform heterozygote advantage (SOS) presented here. Indeed, under both alternative selection models and for all loci, the Overlapping scenario is the most likely and the Southern route scenario is (almost always significantly) the less supported (see Additional file 4: Table S4 and Additional file 5: Table S5).

### North-south genetic variation

The genetic differences between NEAs and SEAs observed in numerous studies are evidenced by both among- and within-population variation. Among populations, significant north-south differentiation can be reproduced under any of the three models but is much more frequently observed under the two-route models (Fig. 3a). By contrast, greater genetic diversity (*H*) within populations in SEA is systematically produced under the Southern-origin model, which is incompatible with the observed data. At this level, differences between NEAs and SEAs can only be explained by the two-route models (Fig. 3b).

### Evolutionary and demographic factors

Under the best of the three models considered, the Overlapping model, we estimated the effects of several evolutionary factors in shaping the genetic patterns. Based on the RMSEP charts, the balancing selection coefficient *s* has a significant impact on the simulated genetic structure (Additional file 6: Table S6). The estimated *s* values are close to each other for HLA−B and HLA−DRB1 under the Overlapping model, with point estimates of 0.79 % (0.12 %-2.2 %) and 0.63 % (0.11 %-2.3 %), respectively (Fig. 4b), while it is only of 0.16 % (0.015 %-1.2 %) for HLA−A. These results are in agreement with the ranking of HLA loci based on balancing selection reported previously [24, 29, 54]. On the other hand, the absolute values that we estimated for selection rates are much lower compared to these studies, where estimated *s* were of 1.5-2.2 %, for HLA−A, 4.2-4.4 %, for HLA−B, and 1.9-2.2 %, for HLA–DRB1, respectively [16, 54-55]. This discrepancy could be due to the fact that we used HLA lineages rather than alleles (used by the other authors), suggesting that natural selection would have left a more visible signature on the latter despite their much more recent origin [32] compared to the long-term evolution of lineages [53, 56]. Alternatively, the observed discrepancy could result from differences in the methods used to estimate selective coefficients, our approach comprising a larger set of parameters including demography, but, on the other hand, no molecular variables. In any case, the selective coefficients estimated for the different HLA loci (regardless of the level of resolution considered to define lineages or alleles) are lower than for other genes known to undergo pathogen-mediated balancing selection, like G6PD/A- (*s* of about 10-20 %) [57] or HbC (4-9 %) [58], or positive directional selection, like Fy*O (6.6 %) [59] or lactase persistence (LP, up to 15.9 %, depending on the studies [60]). The present results suggest that (at least for HLA lineages) the impact of demographic factors on the East Asian HLA genetic variation overcame to a large extent the impact of balancing selection, as also revealed at the global scale by significant correlations between HLA population differentiations and geography resulting from population migrations [29]. Also, the demographic effect was particularly stronger on HLA−A, as supported by lower compatibility (*p-value*) of the Overlapping model at this locus compared to HLA−B and −DRB1 with original statistics (Table 4) and 6 PLS. The compatibility at HLA−A can only be improved when the effect of balancing selection is largely excluded by using less than 5 PLS (Additional file 6: Table S6). This hypothesis is congruent with previous results indicating a nearly neutral mode of evolution for HLA−A [23, 24], with a lower genetic diversity of this gene compared to HLA−B and −DRB1 in different continents [29]. In East Asian populations, for example, some HLA−A lineages are very common, such as A*02 (>20 %), A*11 (>30 % in SEA) and A*24 (>20 % in NEA). By contrast, a higher genetic diversity is maintained at HLA−B and −DRB1 loci [10].

Of course, the selection coefficients estimated here are appropriate only for the selection model with which they have been estimated and could be quite different if HLA genes were affected by selective pressures varying in time and space, in link with environmental changes. The implementation of such complex selective pressures would involve many more assumptions and parameters (range and variance of selection coefficients, spatial patterns of selection, choice of association between selection values and specific HLA alleles) and would constitute an exciting but difficult further development of this study. However, we believe that the three selection models investigated here constitute good approximations for representing the average effect of balancing selection acting on HLA and we showed that the model comparison is robust to the selection model.

For the three HLA loci under study, *Nm* is considerably higher in NEA (between 208 and 489) than in SEA (between 67 and 116), even if the highest probability density (HPD) intervals are wide and partially overlapping (Fig. 4d,f). By treating separately *N* and *m*, we notice that *m* is the decisive factor explaining this different *Nm* (Additional file 6: Table S6). The estimated *m* values for SEA are much lower compared to NEA (Fig. 4e,g), supporting the hypothesis, proposed earlier, that modern humans colonizing East Asia via the Southern route underwent significant founder effects, or passed through periods of isolation with very rapid genetic drift [10]. Human migrations may have been more limited due to the mountainous reliefs of SEA, while long-range migrations in NEA would have been favored by the open landscapes of vast plateaus and plains. As a consequence, a greater differentiation among populations is now observed in SEA: the populations speak languages belonging to many distinct language families (Sino-Tibetan, Miao-Yao, Tai-Kadai and Austro-Asiatic), and are highly differentiated genetically within each of these families [10]; by contrast, in NEA the Altaic- and Mandarin-speaking populations are fairly homogeneous from a genetic point of view [10].

To summarize, despite significant but different effects of balancing selection acting on HLA−A, −B and −DRB1, similar conclusions are drawn for these three HLA loci concerning the likelihood of the different scenarios tested, suggesting that demography shaped the observed genetic patterns to a greater extent than did selection. These results support the hypothesis that the intensity of natural selection which acted on these HLA loci was too weak to blur the main signals of past demographic events of East Asia. Similar observations have also been found in Europe [23].

### Genetic boundaries

We tested the existence and location of two geographic areas, hereafter named “genetic boundaries”, across which populations underwent low gene flow in the past. They could have represented areas where populations originating from different routes came into contact and exchanged few migrants, or where a significant genetic divergence appeared among related populations due to some specific reasons. We first considered, as a potential boundary, the 29-33°N zone, which represents an area between the 0 °C isotherm in winter (Qinling-Huaihe line) and an important geographic barrier (Yangtze River). Indeed, according to several studies using different genetic markers, the current genetic boundary between NEAs and SEAs was located in this zone [10, 61-62]. Actually, the Overlapping model suggests that this boundary, regardless of its precise location, had been formed more recently by the creation of political subdivisions, while a more ancient boundary was located more to the north [10]. The high likelihood obtained in the present study for this model strongly supports the existence of an ancient boundary, in NEA (41-45°N zone), between the populations originating from the two migration routes, and the emergence, at a more recent time, of a genetic boundary around Qinling-Huaihe line or Yangtze River (29-33°N zone). The latter may have been due to the political subdivisions coinciding with the diversification of Chinese languages [37]. It is possible that, during a long period in the last Ice Age, the Mongolian Plateau and Siberia were not conducive to Southern populations (coming from the Southern route) practicing hunting and gathering in tropical and subtropical environments, whereas populations from the Northern route were better adapted to these harsh environments [63]. The separation between these populations would have thus been related to different degrees of cultural adaptation to their environment rather than to geographical barriers such as mountain ranges.

### Long-term genetic exchanges among NEAs and SEAs

The admixture time *T* measures the duration of genetic exchange between NEAs and SEAs since they met in East Asia. Under the Overlapping model, *T* appears as a critical parameter (Additional file 6: Table S6), and the most likely values estimated for *T* are very consistent among the three HLA loci (in generations: 1,213 for HLA−A, 1,193 for HLA−B and 1,363 for HLA−DRB1, Fig. 4c), corresponding to about 24,000-36,000 years depending on the generation time considered (20-30 years). The posterior distributions for the three estimated values are relatively large, but agree among the loci (95 % HPD Interval: 217-2,219 for HLA−A, 200-2,167 for HLA−B and 290-2,276 for HLA−DRB1, Fig. 4c). Nevertheless, even the lower limits of these ranges are greater than 4,000 years, reflecting that a short-term genetic exchange is very unlikely to have created the existing genetic structure. These results contradict the assumption of some scholars who explain the observed genetic patterns by recent gene flow from Northwest Asia through the Silk Road, about only 2,000 years ago [3, 5]. Actually, they suggest a longer and more extensive overlap between NEA and SEA populations than estimated so far with neutral data, which may either reflect a stronger influence of a Northern route that is not captured by these neutral data or extensive geographic differences in pathogen selection at HLA genes that is not captured by the selection models used in our study.

For reasons of simplification, we decided to simulate the human expansions by the two routes simultaneously, but our results do not exclude that the Southern route was older, as suggested by some genetic [5, 64] and archaeological [65] data. However, the *T* values that we estimated imply that human migrations via the Northern route date back at least to the Neolithic, and more probably to the Upper Paleolithic. The genetic admixture between the NEAs and SEAs may have initiated just after, or even before the Last Glacial Maximum (23-21 kya) [66]. Genetic differences between the early populations originating from the two migration routes would be, to some extent, reflected by the recent analyses of ancient human DNA from Tianyuan Cave (~40 kya) in northern China [67], on one side, and from Mal’ta (~24 kya) and Afontova Gora-2 (~17 kya) in Southern Siberia [68], on the other side (Fig. 1 and Additional file 2: Table S2). Also, the high degree of morphological diversity observed between individuals found in the Upper cave (~18 kya) [69], several kilometers from Tianyuan cave, would be explained by early contact of different populations in NEA, as suggested by the Overlapping model. This scenario is further consistent, on a larger geographical scale, to a model of multiple dispersals Out-of-Africa recently tested and supported by genomic and cranial phenotype data [70].

## Conclusions

In this study, we simulated different scenarios of human peopling history in East Asia, i.e. the Southern-origin model (one-route), the Pincer model (two-route) and the Overlapping model (also two-route). Our quantitative analyses of 100,000 simulations for each model show that the Southern-origin model is virtually unable to reproduce genetic data compatible with the observations. The two-route models taking into account human colonization into the region via both a Southern and a Northern route are significantly more likely. Moreover, the results are in favor of the Overlapping model compared to the Pincer model.

In addition, our study reveals that evolutionary mechanisms related to the demographic history of East Asian populations had major effects on the observed genetic patterns compared to natural selection acting on HLA, which is weak for the three HLA loci (and especially so for HLA−A). In particular, we found a significantly lower migration rate among populations in SEA than in NEA, suggesting that SEAs underwent severe bottlenecks or periods of substantial isolation, likely due to the mountainous reliefs of this region. Our results also indicate the emergence of a genetic boundary characterized by limited gene flow between NEAs and SEAs around the Qinling-Huaihe line or Yangtze river (29-33°N zone), representing a limit of an “Altaicization” of northern Chinese languages proposed by some linguists [71] and likely related to imperial age political subdivisions, while such a boundary would have existed further north (41-45°N zone) in a more remote period. Finally, the genetic exchanges between NEAs and SEAs, which took place when the two migration routes overlapped in East Asia, probably lasted a very long time which may trace back at least to the Neolithic but more probably to the Upper Paleolithic. All these events eventually gave rise to the pronounced but continuous HLA genetic variation currently observed between Northern and Southern East Asian populations.

## Availability of supporting data

The references of all genetic data used in this study are given as supporting Additional file 1: Table S1. The program SELECTOR is freely available at http://ua.unige.ch/en/agp/tools/selector/.

## Additional files

Additional file 1: Table S1. Information of populations sampled for HLA−A, −B, −DRB1 loci and cited in this study. (PDF 81 kb) Additional file 2: Table S2. Information of majorModel comparison using the Latitudinal Overdominant Selection model (LOS Upper Paleolithic archaeological sites with human remains in East Asia. (PDF 87 kb) Additional file 3: Table S3. Distribution of simulated, retained and observed values of each pair of statistics for each HLA locus. (PDF 1316 kb) Additional file 4: Table S4. Model comparison using the Latitudinal Overdominant Selection model (LOS). (PDF 59 kb) Additional file 5: Table S5. Model comparison using the Negative Frequency-Dependent Selection model (NFDS). (PDF 75 kb) Additional file 6: Table S6. RMSEP charts of the parameters under the Overlapping model. (PDF 158 kb) Additional file 7: Table S7. Results of Kolmogorov-Smirnov test against a uniform distribution. (PDF 115 kb)

## Abbreviations

ABC: approximate Bayesian computation
HLA: human leukocyte antigen
LOS: latitudinal overdominant selection
NEA: North of East Asia
NEAs: Northern East Asian populations
NFDS: negative frequency-dependent selection
PLS: partial least squares
RMSEP: root mean squared error prediction
SEA: South East Asia
SEAs: Southern East Asian populations
SNPs: single nucleotide polymorphisms
SOS: symmetric overdominant selection
STRs: short tandem repeats
