# Supplementary material for: Computer simulation of human leukocyte antigen genes supports two main routes of colonization by human populations in East Asia
Source: BMC Evol Biol. 2015 Nov 4;15:240. doi: 10.1186/s12862-015-0512-0 (PMC4632674; doi:10.1186/s12862-015-0512-0)
Supplement: Additional file 1: Table S1. — Information of populations sampled for HLA−A, −B, −DRB1 loci and cited in this study. (PDF 81 kb) [file 12862_2015_512_MOESM1_ESM.pdf]

Table S1 Information of populations sampled for HLA-A, -B, -DRB1 loci and cited in this study

No. in Figure 1	Population	Sample size	Location	Linguistic group	Year	Reference
**1**	Tuvinians	188	52°N 93°E	Altaic	2002	[1]
**2**	Oold Mongols	52	47°N 95°E	Altaic	2003	[2]
**3**	Khalkha Mongols	100	48°N 107°E	Altaic	2003	[2]
**4**	Southern Mongols	106	41°N 112°E	Altaic	2008	[3]
**5**	Liaoning Han	9678	41°N 123°E	Sino-Tibetan	2005	[4]
**6**	Shanxi Han	7440	38°N 113°E	Sino-Tibetan	2005	[5]
**7**	Shandong Han	7418	37°N 117°E	Sino-Tibetan	2006	[6]
**8**	Xi'an Han	7016	34°N 109°E	Sino-Tibetan	2005	[7]
**9**	Henan Han	3874	33°N 114°E	Sino-Tibetan	2011	[8]
**10**	Anhui Han	2816	32°N 117°E	Sino-Tibetan	2005	[9]
**11**	Hubei Han	4026	31°N 114°E	Sino-Tibetan	2006	[10]
**12**	Zhejiang Han	1100	29°N 121°E	Sino-Tibetan	2010	[11]
**13**	Hunan Han	3664	28°N 113°E	Sino-Tibetan	2006	[12]
**14**	Jiangxi Han	2210	29°N 116°E	Sino-Tibetan	2006	[13]
**15**	Fujian Han	171	25°N 118°E	Sino-Tibetan	2004	[14]
**16**	Maonan	108	25°N 108°E	Tai-Kadai	2007	[15]
**17**	Guangdong Han	406	23°N 113°E	Sino-Tibetan	1999	[16]
**18**	Zhuang	104	23°N 107°E	Tai-Kadai	2011	[17]
**19**	Muong	107	21°N 105°E	Austro-Asiatic	2006	[18, 19]

References

1. Martinez-Laso J, Sartakova M, Allende L, Konenkov V, Moscoso J, Silvera-Redondo C et al. HLA molecular markers in Tuvinians: a population with both Oriental and Caucasoid characteristics. Ann Hum Genet. 2001;65(Pt 3):245-261.
2. Machulla HK, Batnasan D, Steinborn F, Uyar FA, Saruhan-Direskeneli G, Oguz FS et al. Genetic affinities among Mongol ethnic groups and their relationship to Turks. Tissue Antigens. 2003;61(4):292-299.
3. Shen CM, Zhu BF, Li SB. [HLA-A, B, and DRB1 gene polymorphisms in Mongol ethnic group of Inner Mongolia, China]. Yi Chuan. 2008;30(2):164-168.
4. Meng QL, Yu WJ, Liang XH, Wang M, Hu RH, Bi XL et al. [HLA gene distribution and haplotype analysis of blood stem cell donors in Liaonan aera]. Zhongguo Shu Xue Za Zhi. 2007;20(1):30-32.
5. Lan T, Zhang DM, Wang GQ. [Polymorphism of HLA-A,-B and DRB1 in Han population of Shanxi province]. Zhong Hua Yi Xue Yi Chuan Xue Za Zhi. 2005;22(5):583-584.
6. Song YH, Li WC, Nie XM, Wang M, Liu Y, Zhang P. [HLA- A, B, DRB1 allele polymorphism in Hans from Shandong province of China]. Yi Xue Jian Yan Yu Lin Chuang. 2006;17(3):16-19.
7. Liu ML, Zhang Y, Liu S. [Study on the polymorphism of HLA-A, B, DR genes of population in Xi'an area, China]. Chinese Journal of Blood Transfusion. 2005;18(6):470-473.
8. Wang Z, Zhang B, Ma R, Du J, Kong D. [HLA-A, B and DR high-resolution gene polymorphism of Henan Stem Cell Registry]. Journal of Clinical Rehabilitative Tissue Engineering Research. 2011;15(31):5801-5804.
9. Gao SQ, Wu GG, Li XM, Cheng LH, Zou HY, Li Z et al. [Characteristic and distribution of human leukocyte antigen-A, B, DRB1 genes and haplotypes in Anhui Chinese Han population]. Lin Chuang Shu Xue Yu Jian Yan. 2005;7(3):161-169.
10. Zhu YY, Yin P, Shen G, Wu JM, Liu GJ, Zou J et al. [Study on polymorphism of HLA-A, B and DRB1 genes in Chinese Han population, Hubei]. Gong Gong Wei Sheng Yu Yu Fang Yi Xue. 2006;17(6):11-13.
11. He J, Zhang W, He Y, Tao S, Han Z, Zhu F et al. [Analysis of high-resolution HLA-A, -B, -DRB1 allelic frequency and haplotypes in Zhejiang Han population]. Chin J Microbiol Immunol. 2010;30(9):800-803.
12. Xie YB, Wang CL, Li S, Xie YX. Study on polymorphism of HLA- A, B, and DRB1 alleles in Hunan population. Practical Preventive Medicine. 2006;13(1):4-7.
13. Cheng LH, Wu GG, Li XM, Gao SQ, Jin SZ, Cheng X et al. [Allele and haplotype frequencies for the loci HLA-A, B and DRB1 in 2210 Jiangxi Chinese Han unrelated bone marrow donors]. Lin Chuang Shu Xue Yu Jian Yan. 2006;8(1):5-12.
14. Huang RX, Pei B, Chen CR, Ni HY, Zhou JJ, Hong SY. [Reverse PCR-SSO technique for HLA polymorphism study in the Han population of Fujian, China]. Zhongguo Shu Xue Za Zhi. 2004;17(5):351-352.
15. Ogata S, Shi L, Matsushita M, Yu L, Huang XQ, Sun H et al. Polymorphisms of human leucocyte antigen genes in Maonan people in China. Tissue Antigens. 2007;69(2):154-160.
16. Xiao LL, Chen HT, Ye X, Ma LY, Tan Y, Zhang S. [Characterization and distribution of HLA polymorphism in the Guangdong Han population]. Zhonghua Wei Sheng Wu Xue He Mian Yi Xue Za Zhi. 1999;19(4):302-305.
17. Shi L, Huang XQ, Shi L, Tao YF, Yao YF, Yu L et al. HLA polymorphism of the Zhuang population reflects the common HLA characteristics among Zhuang-Dong language-speaking populations. Journal of Zhejiang University Science B. 2011;12(6):428-435.
18. Busson M, Vu Trieu A, Labelle P, Pham-Van K, Ho-Quang H, Bouteiller AM et al. HLA-DRB1 and DQB1 allele distribution in the Muong population exposed to malaria in Vietnam. Tissue Antigens. 2002;59(6):470-474.
19. Riccio M-E. Etude des polymorphismes génétiques des systèmes HLA, GM, ADN mitochondrial et chromosome Y dans les populations vietnamiennes. Geneva: University of Geneva; 2015.
