# Supplementary material for: Computer simulation of human leukocyte antigen genes supports two main routes of colonization by human populations in East Asia
Source: BMC Evol Biol. 2015 Nov 4;15:240. doi: 10.1186/s12862-015-0512-0 (PMC4632674; doi:10.1186/s12862-015-0512-0)
Supplement: Additional file 2: Table S2. — Information of majorModel comparison using the Latitudinal Overdominant Selection model (LOS Upper Paleolithic archaeological sites with human remains in East Asia. (PDF 87 kb) [file 12862_2015_512_MOESM2_ESM.pdf]

Table S2 Information of major Upper Paleolithic archaeological sites with human remains in East Asia

ID in Figure 1	Name	Location	*Homo sapiens* remains	Dating (kya)	Reference
**a**	Mal'ta	52.9°N 103.5°E	Much of a cranium, parts of the mandible and maxilla, several vertebrae and ribs, one humerus, fragment of a lower limb bone, two phalanges, fragments of two femora, fragments of two tibiae and a second set of teeth	~24	[1]
**b**	Afontova Gova	56.0°N 93.0°E	One premolar, fragments of a left radius, ulna, humerus, a phalanx, and part of the frontal	~17	[1]
**c**	Upper Cave	39.7°N 115.9°E	Three well preserved skulls, one cranium and some pelvic and femur bones	~18	[2]
**d**	Tianyuan Cave	39.7°N 115.9°E	34 fragments including the anterior and right side of a mandible, two sternal segments, both scapulae, both humeri, an ulna, a radius, three carpals, five manual phalanges, both femora and tibiae, a distal fibula, a talus, a calcaneus, four metatarsals, and two pedal phalanges	42-39	[3]
**e**	Liujiang	24.2°N 109.4°E	One almost complete skull and several pieces of postcranial bones, exact depth ambiguous	> 67	[4]
**f**	Chochen	23.1°N 120.4°E	Two teeth and seven cranial fragments	30-20	[5]
**g**	Minatogawa	26.1°N 127.8°E	Three skeletons	~18	[6]
**h**	Moh Khiew Cave	8.4°N 98.9°E	One skeleton	~26	[7]

References

1. Raghavan M, Skoglund P, Graf KE, Metspalu M, Albrechtsen A, Moltke I et al. Upper Palaeolithic Siberian genome reveals dual ancestry of Native Americans. Nature. 2014;505(7481):87-91.
2. An Z. Radiocarbon dating and the prehistoric archaeology of China. World Archaeol. 1991;23:193-200.
3. Shang H, Tong H, Zhang S, Chen F, Trinkaus E. An early modern human from Tianyuan Cave, Zhoukoudian, China. Proc Natl Acad Sci U S A. 2007;104(16):6573-6578.
4. Shen G, Wang W, Wang Q, Zhao J, Collerson K, Zhou C et al. U-Series dating of Liujiang hominid site in Guangxi, Southern China. Journal of human evolution. 2002;43(6):817-829.
5. Shikama T, Ling CC, Shimoda N, Baba H. Discovery of fossil Homo sapiens from Chochen in Taiwan. J Anthrop Soc Nippon. 1976;84(2):131-138.
6. Kobayashi H, Hirose T, Sugino M, Watanabe N. University of tokyo radiocarbon measurements V. Radiocarbon. 1974;16(3):381-387.
7. Matsumura H, Pookajorn S. A morphometric analysis of the Late Pleistocene Human Skeleton from the Moh Khiew Cave in Thailand. Homo : internationale Zeitschrift fur die vergleichende Forschung am Menschen. 2005;56(2):93-118.
