# Supplementary material for: Computer simulation of human leukocyte antigen genes supports two main routes of colonization by human populations in East Asia
Source: BMC Evol Biol. 2015 Nov 4;15:240. doi: 10.1186/s12862-015-0512-0 (PMC4632674; doi:10.1186/s12862-015-0512-0)
Supplement: Additional file 3: Table S3. — Distribution of simulated, retained and observed values of each pair of statistics for each HLA locus. (PDF 1316 kb) [file 12862_2015_512_MOESM3_ESM.pdf]

Table S3 Distribution of simulated, retained and observed values of each pair of statistics for each HLA locus. The simulated data have been generated under the Overlapping model and are presented by black dots, while the retained and observed data are plotted by blue dots and red asterisk, respectively

HLA-A	HLA-B	HLA-DRB1
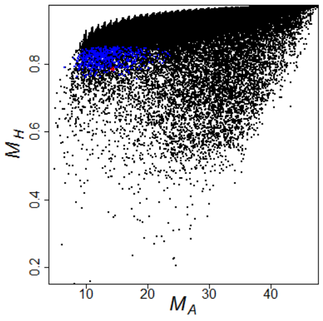	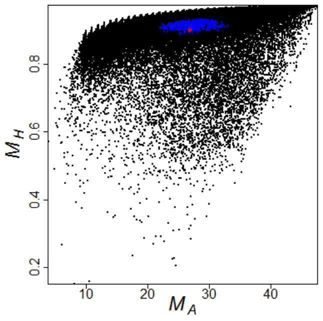	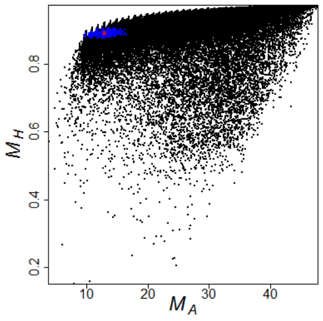
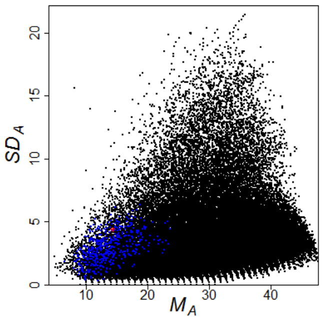	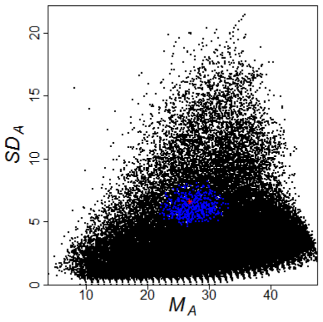	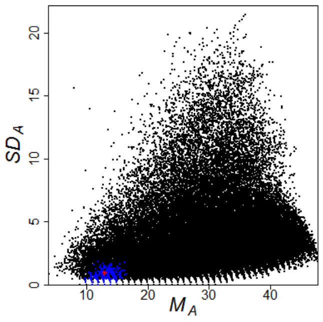
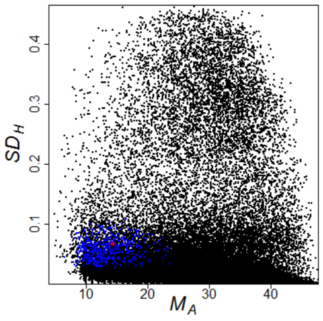	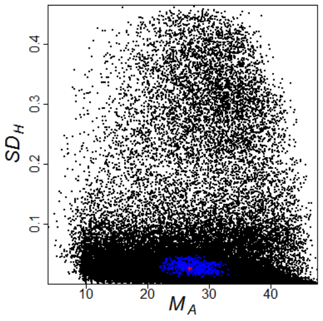	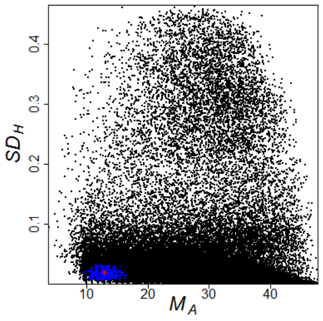
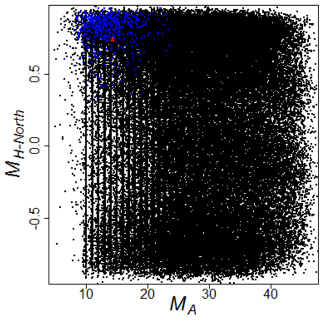	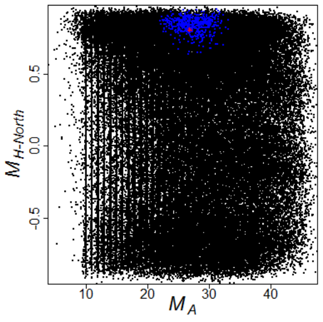	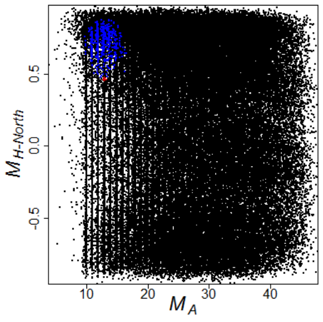

HLA-A	HLA-B	HLA-DRB1

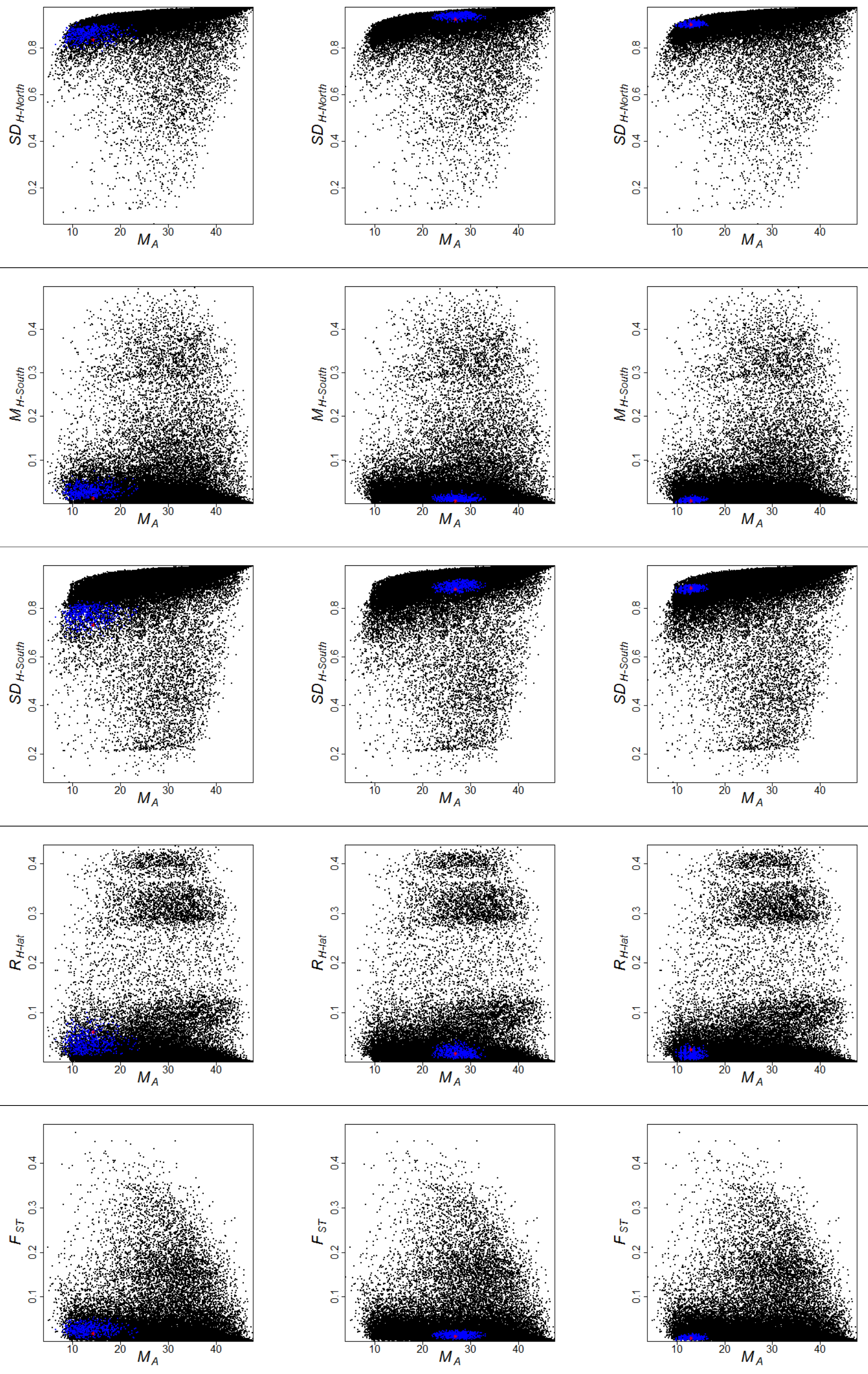

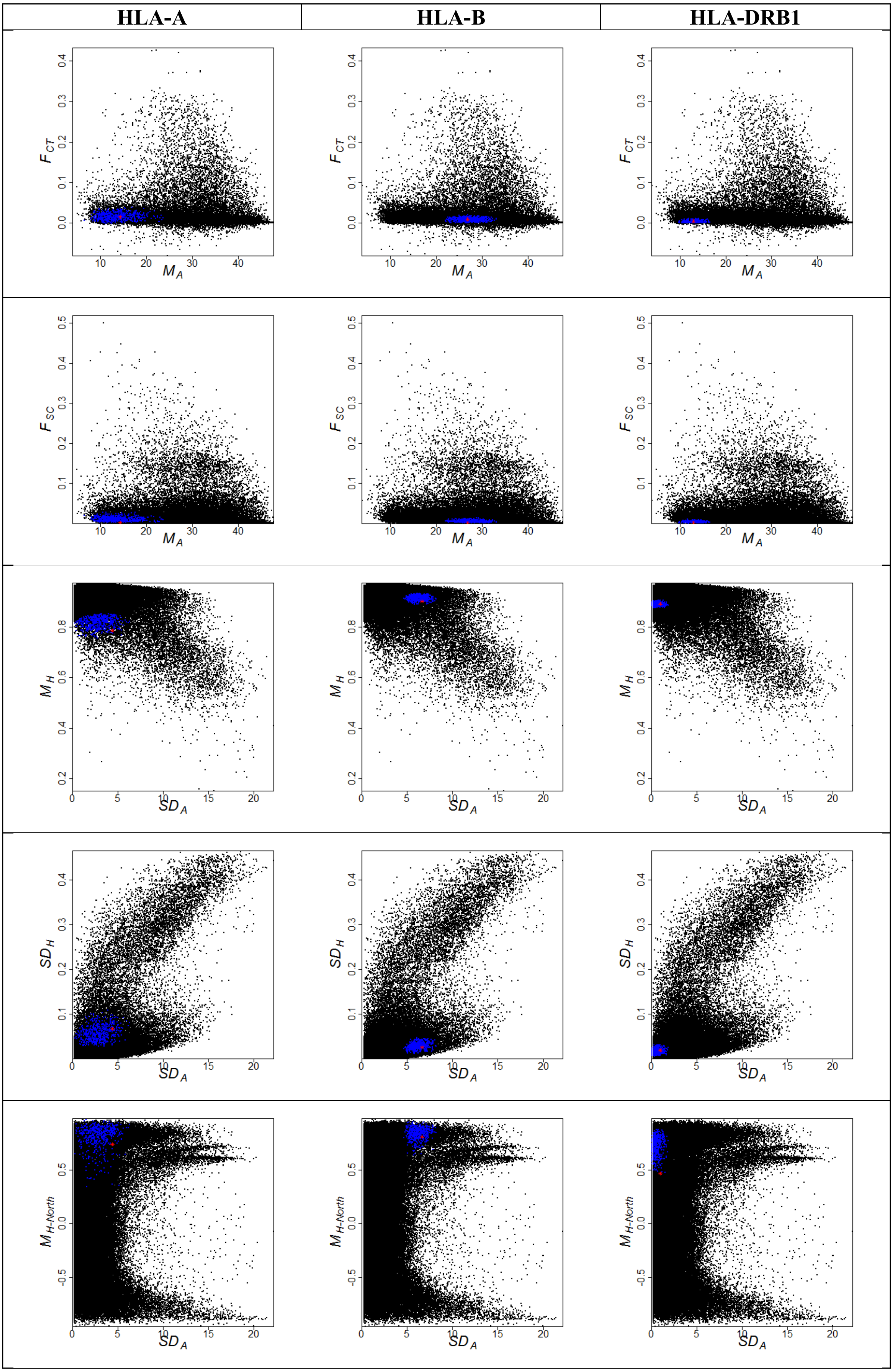
HLA-A
HLA-B
HLA-DRB1
F CT
M A
F SC
M H
SD A
SD H
M H-North

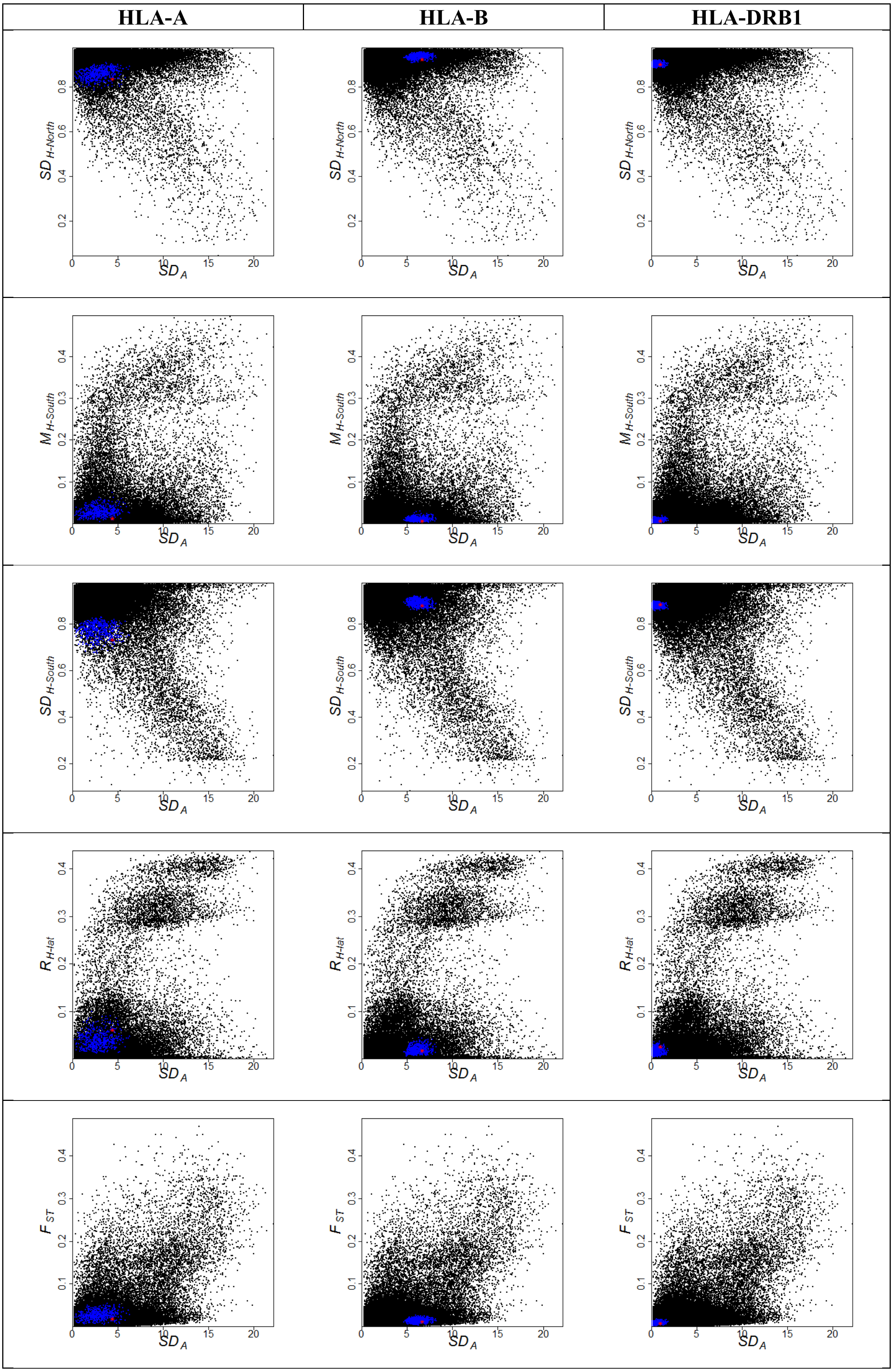
HLA-A
HLA-B
HLA-DRB1
SD A
SD H-North
M H-South
SD H-South
R H-lat
F ST

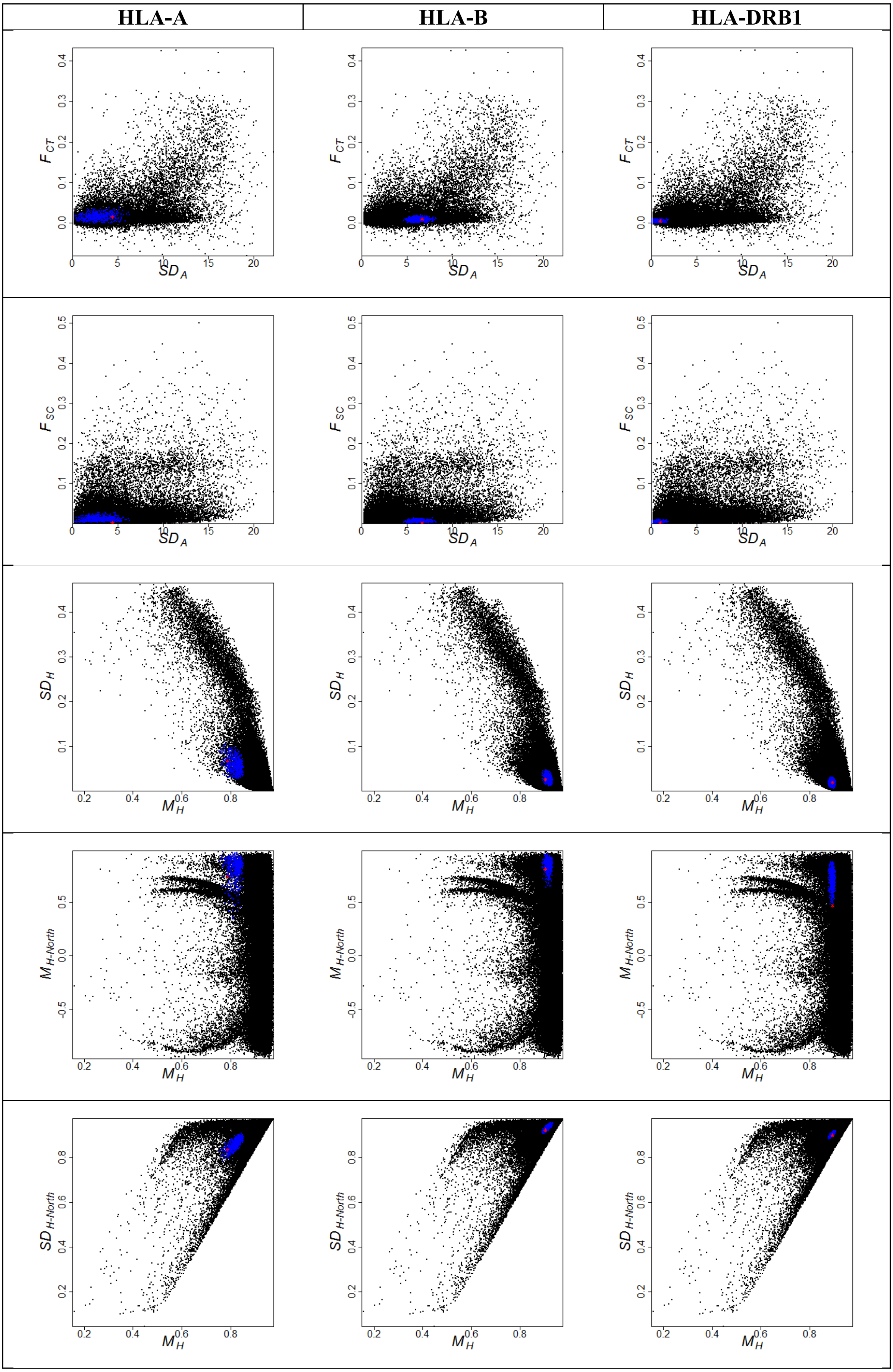
HLA-A
HLA-B
HLA-DRB1
F_CT
SD_A
F_SC
SD_H
M_H
M_H-North
SD_H-North

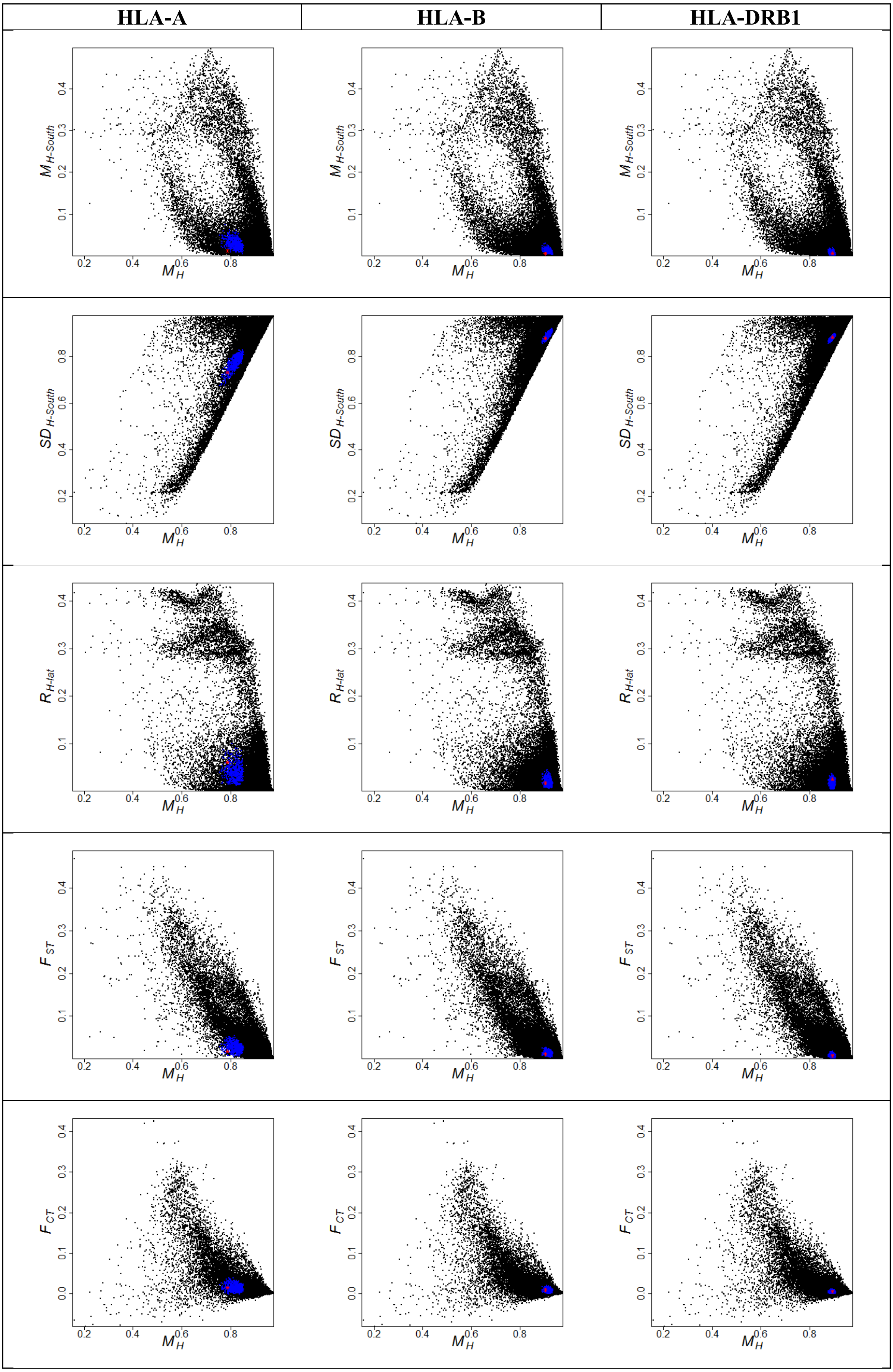
HLA-A
HLA-B
HLA-DRB1
M H-South
SD H-South
R H-lat
F ST
F CT
M H
0.2
0.4
0.6
0.8
0.0
0.1
0.2
0.3
0.4

HLA-A	HLA-B	HLA-DRB1

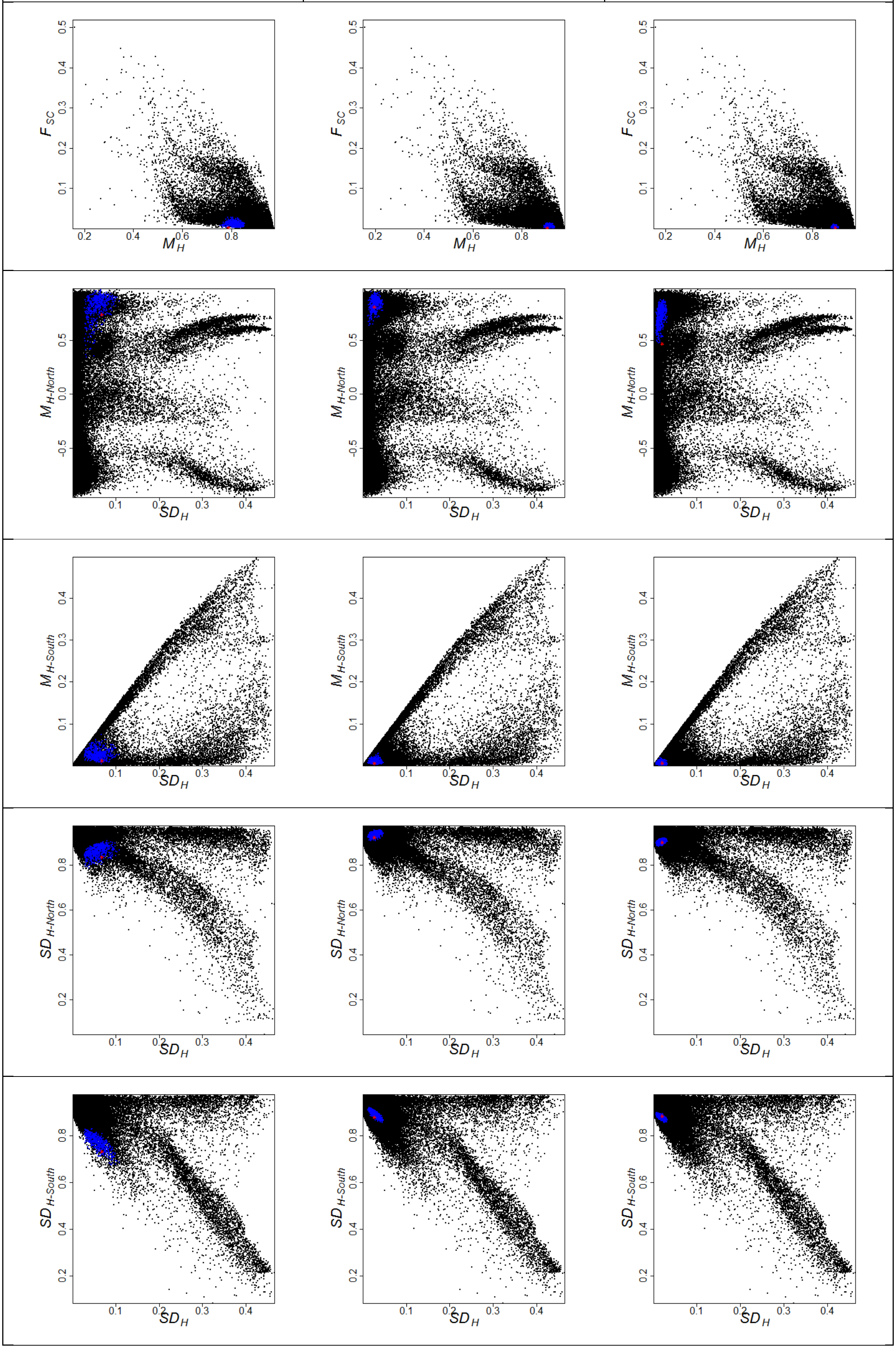

HLA-A	HLA-B	HLA-DRB1

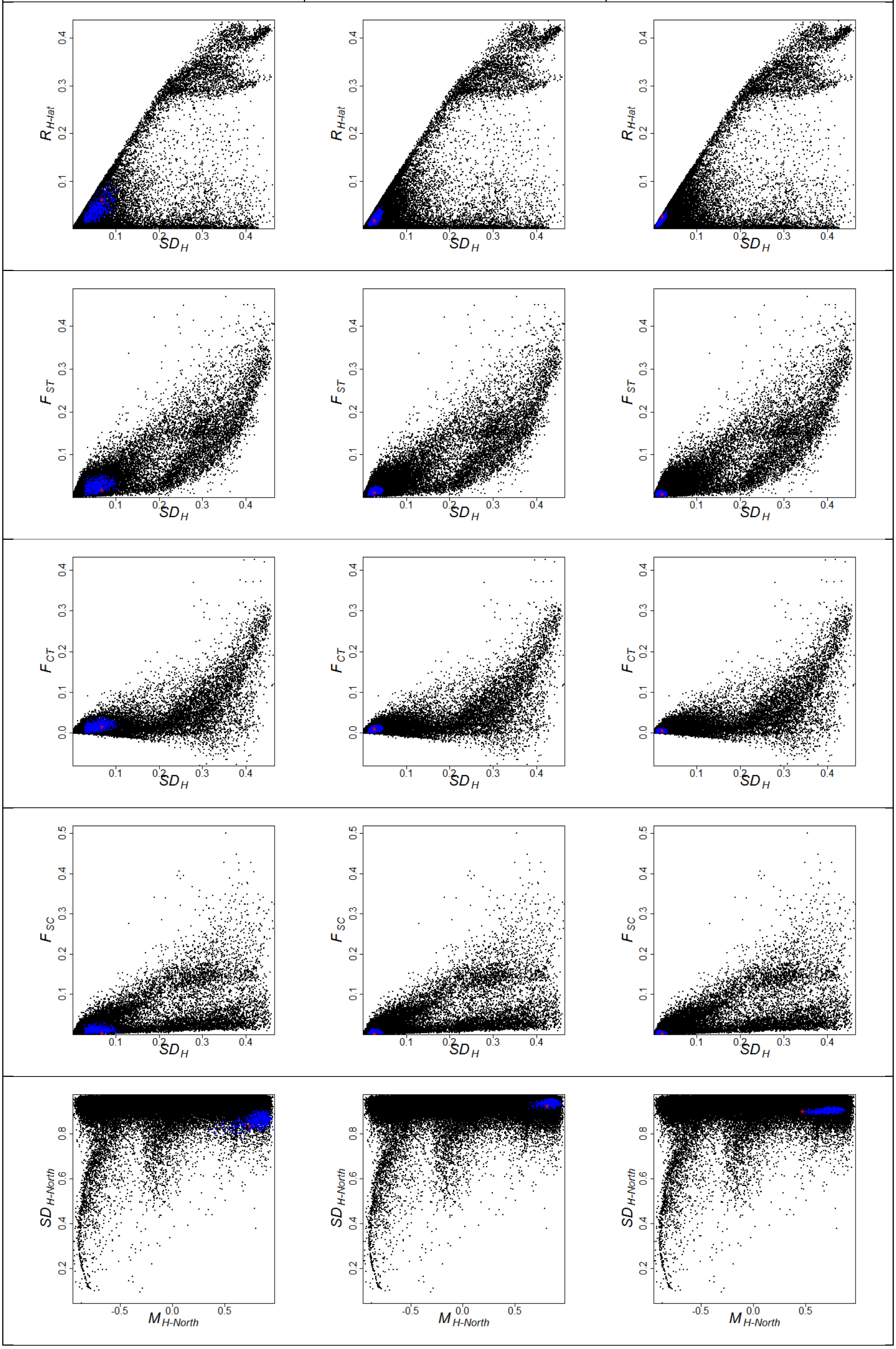

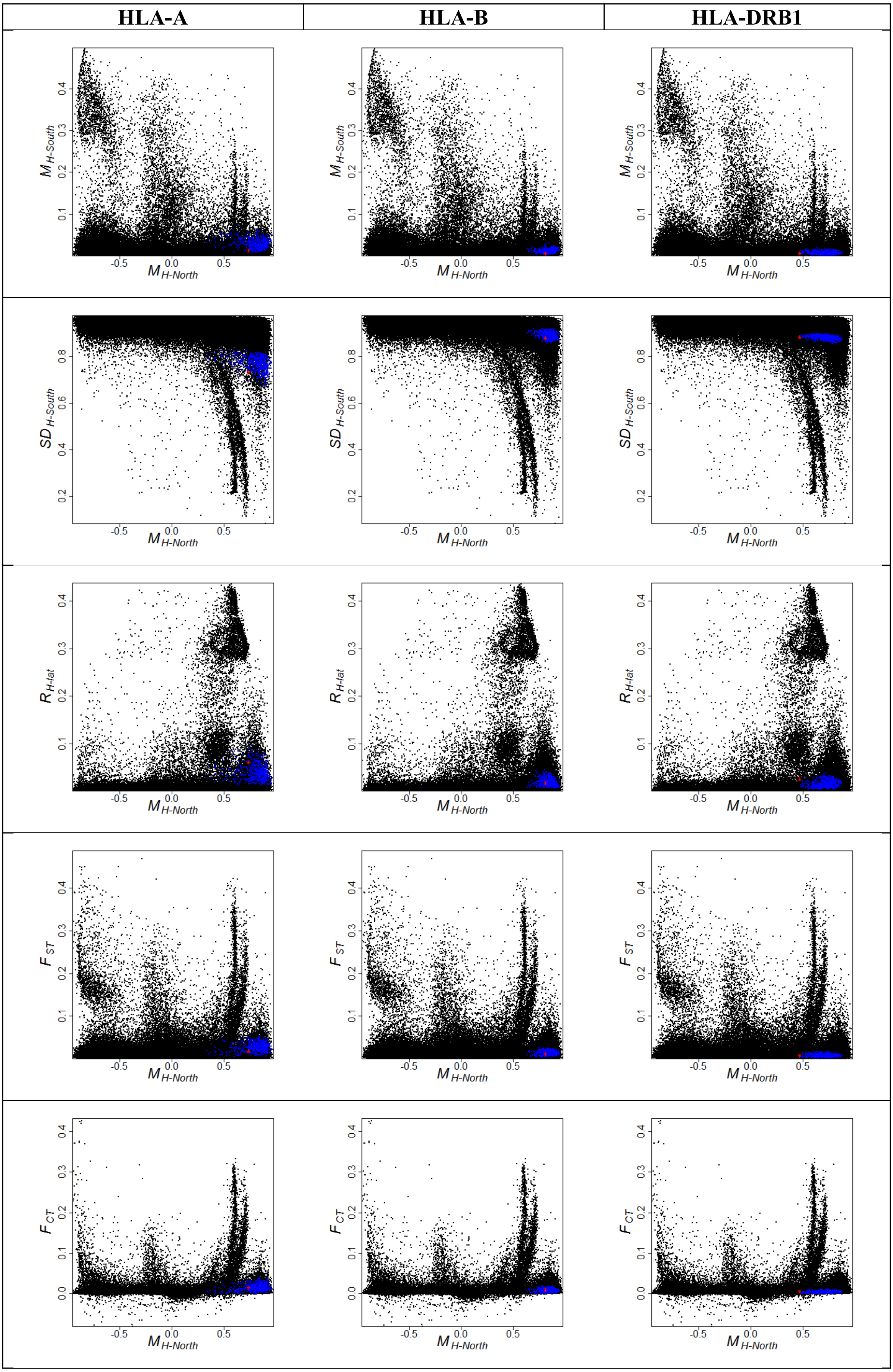

HLA-A
HLA-B
HLA-DRB1
$M_{H\text{-}South}$
$SD_{H\text{-}South}$
$R_{H\text{-}lat}$
F_{ST}
F_{CT}
$M_{H\text{-}North}$

HLA-A	HLA-B	HLA-DRB1

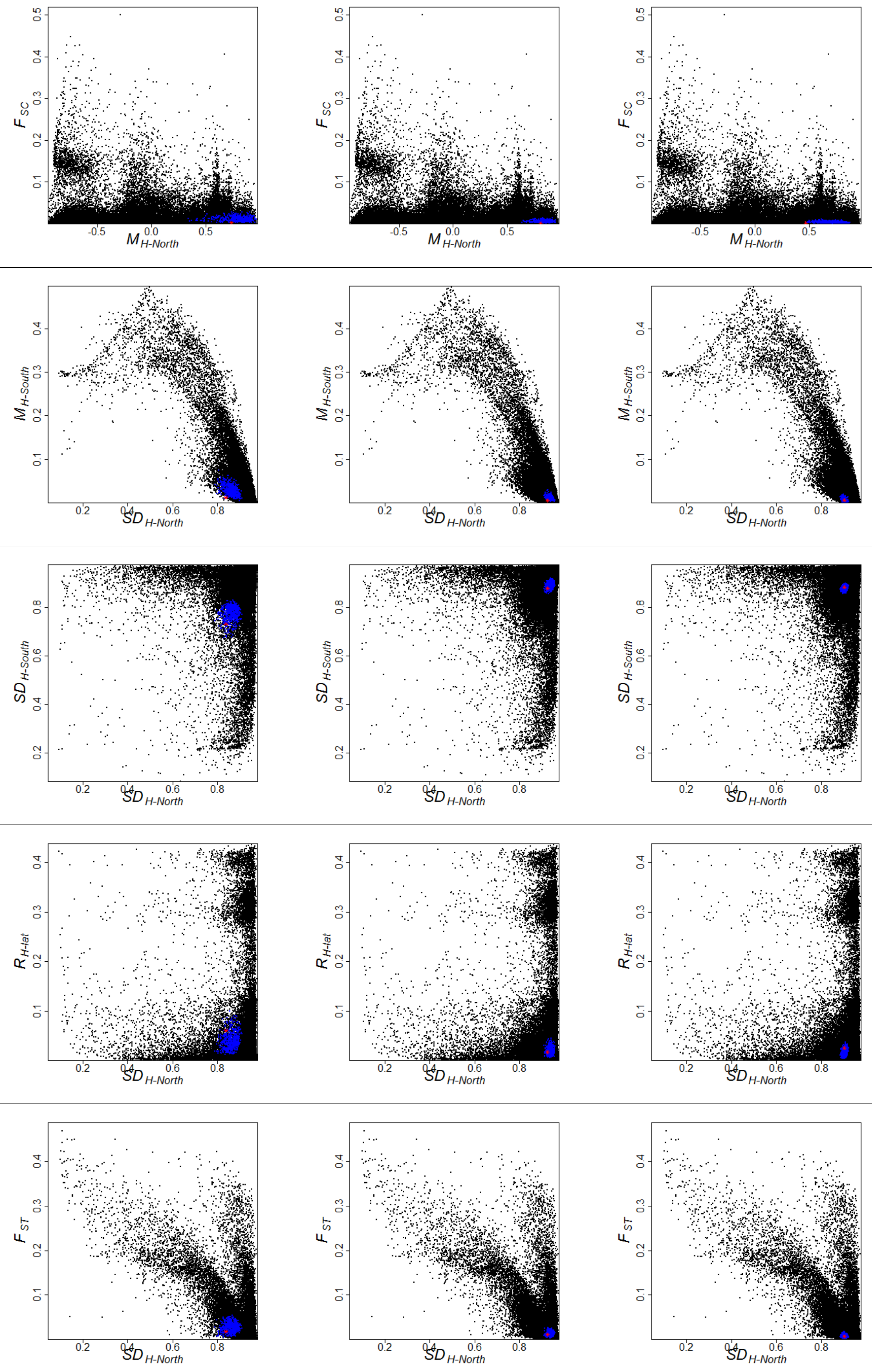

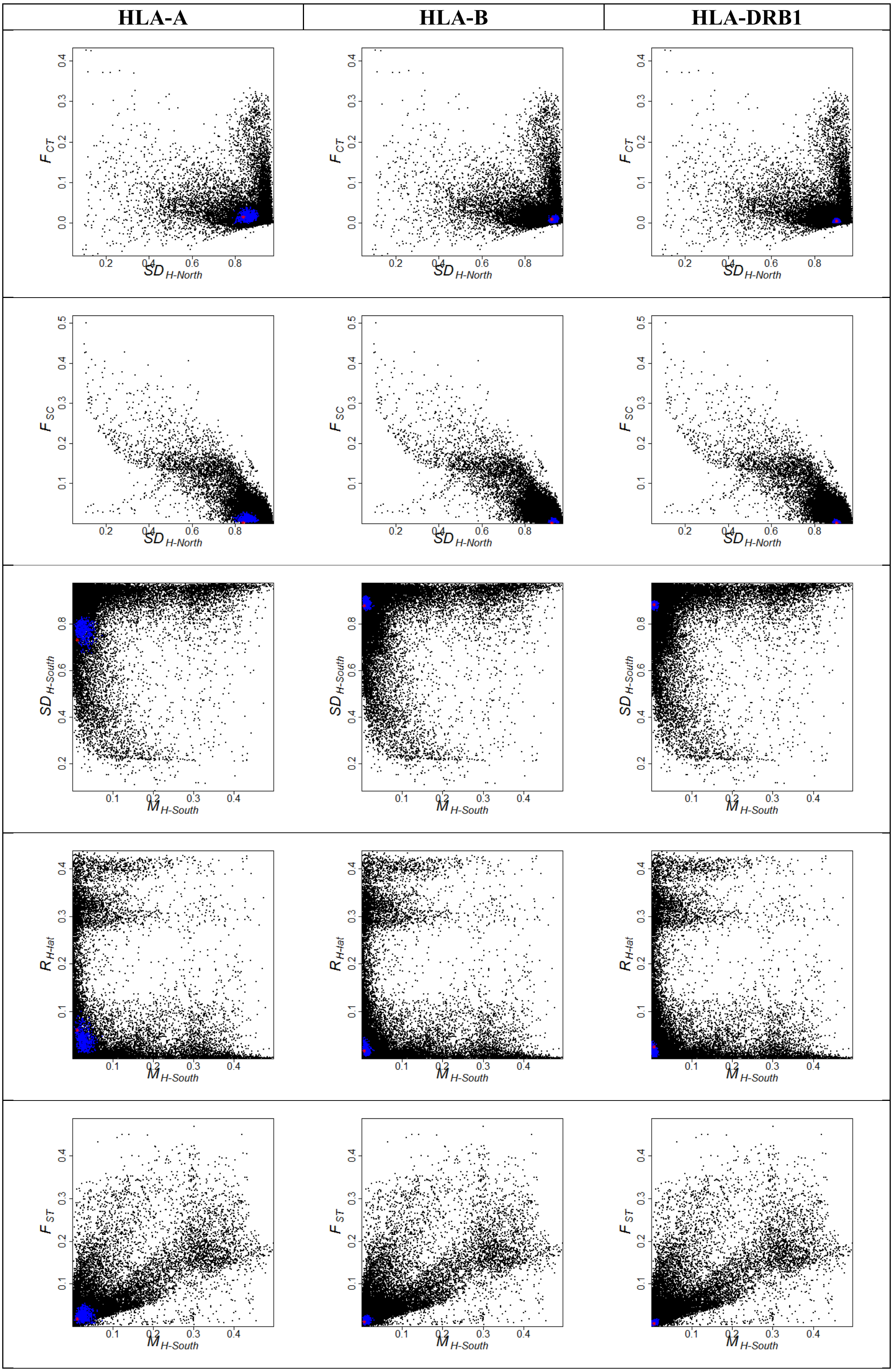
HLA-A
HLA-B
HLA-DRB1
F CT
SD H-North
F SC
SD H-South
M H-South
R H-lat
F ST

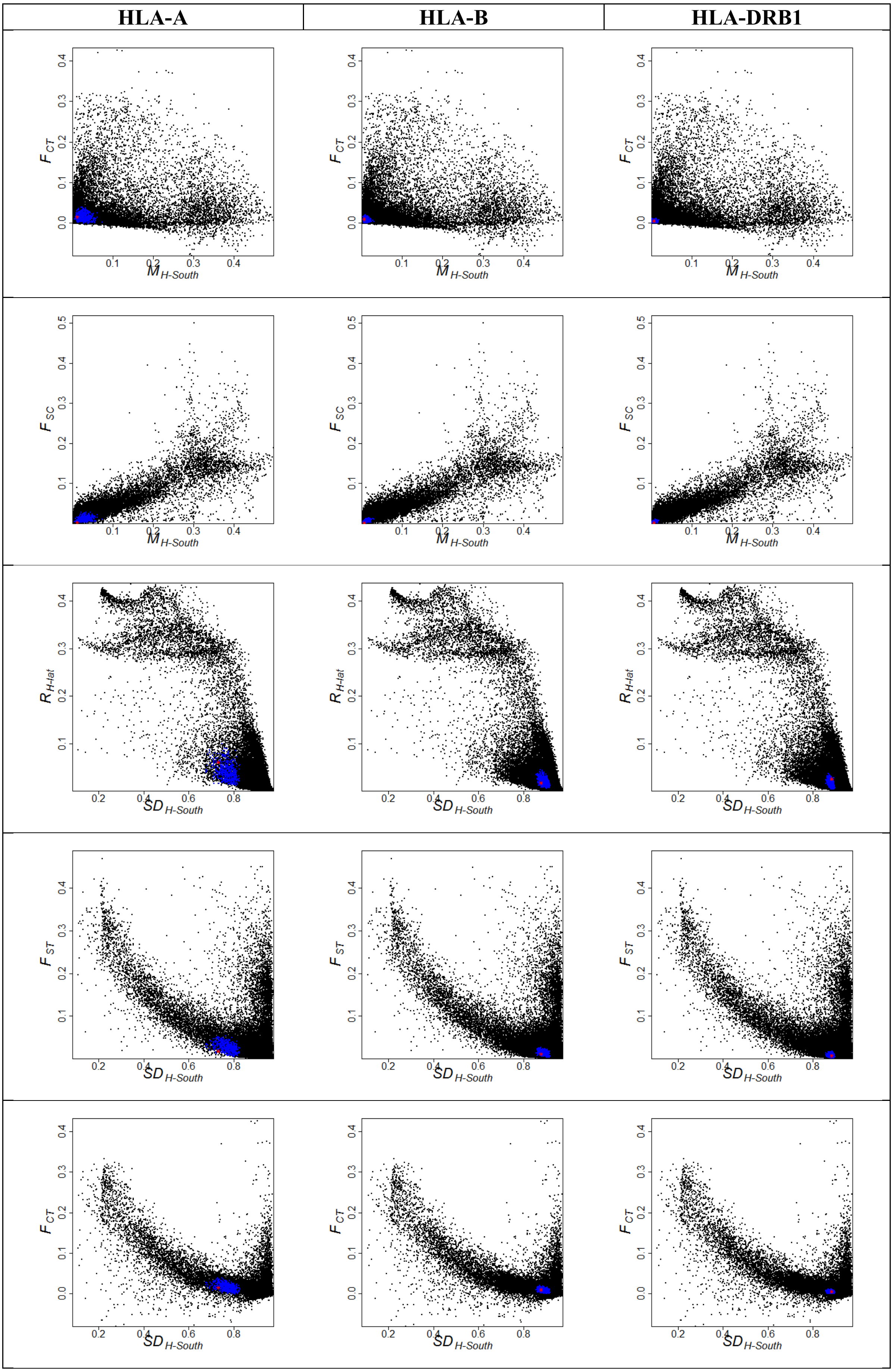
HLA-A
HLA-B
HLA-DRB1
F CT
M H-South
F SC
R H-lat
SD H-South
F ST

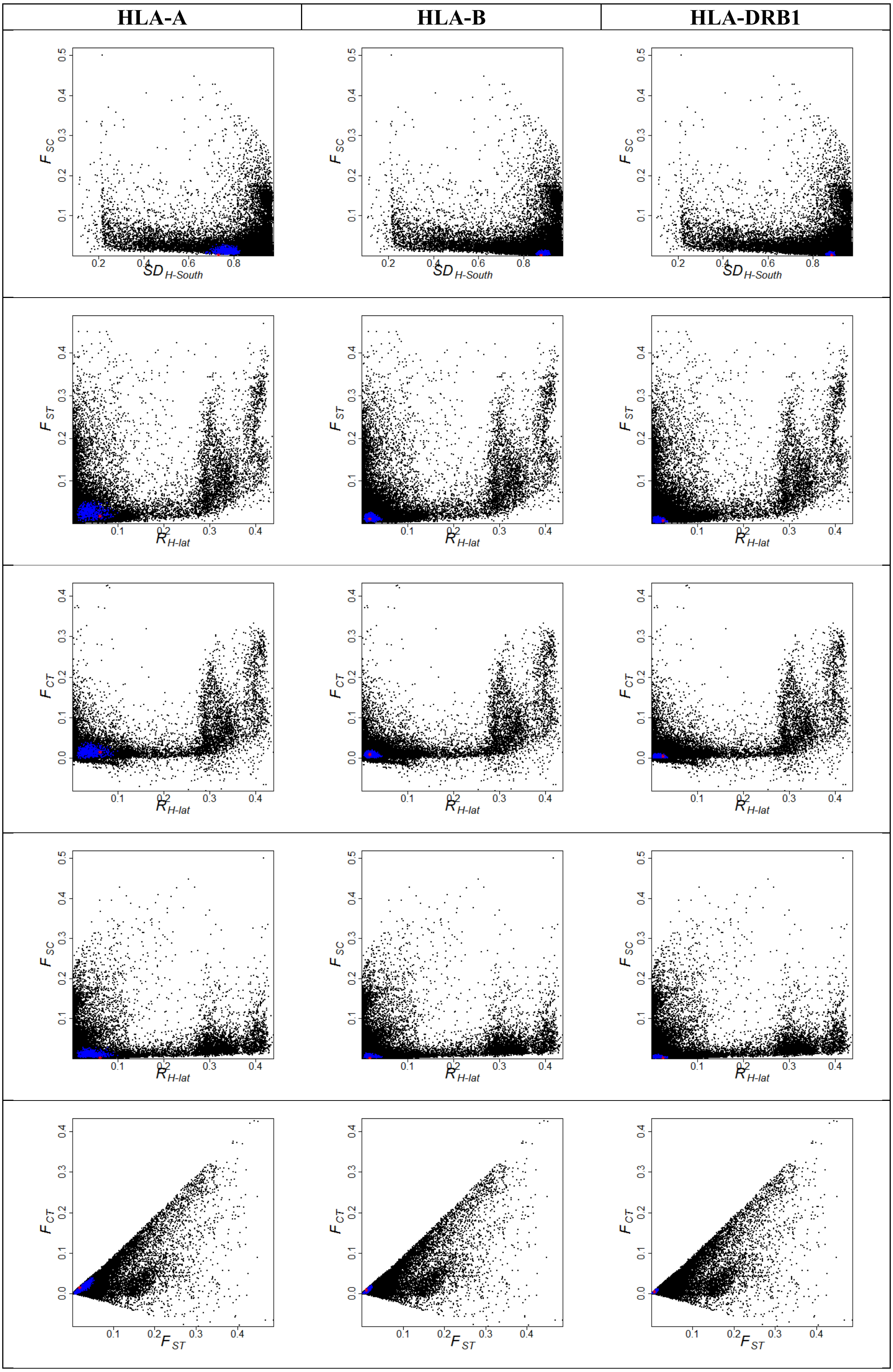
HLA-A
HLA-B
HLA-DRB1
F SC
SD H-South
F ST
R H-lat
F CT

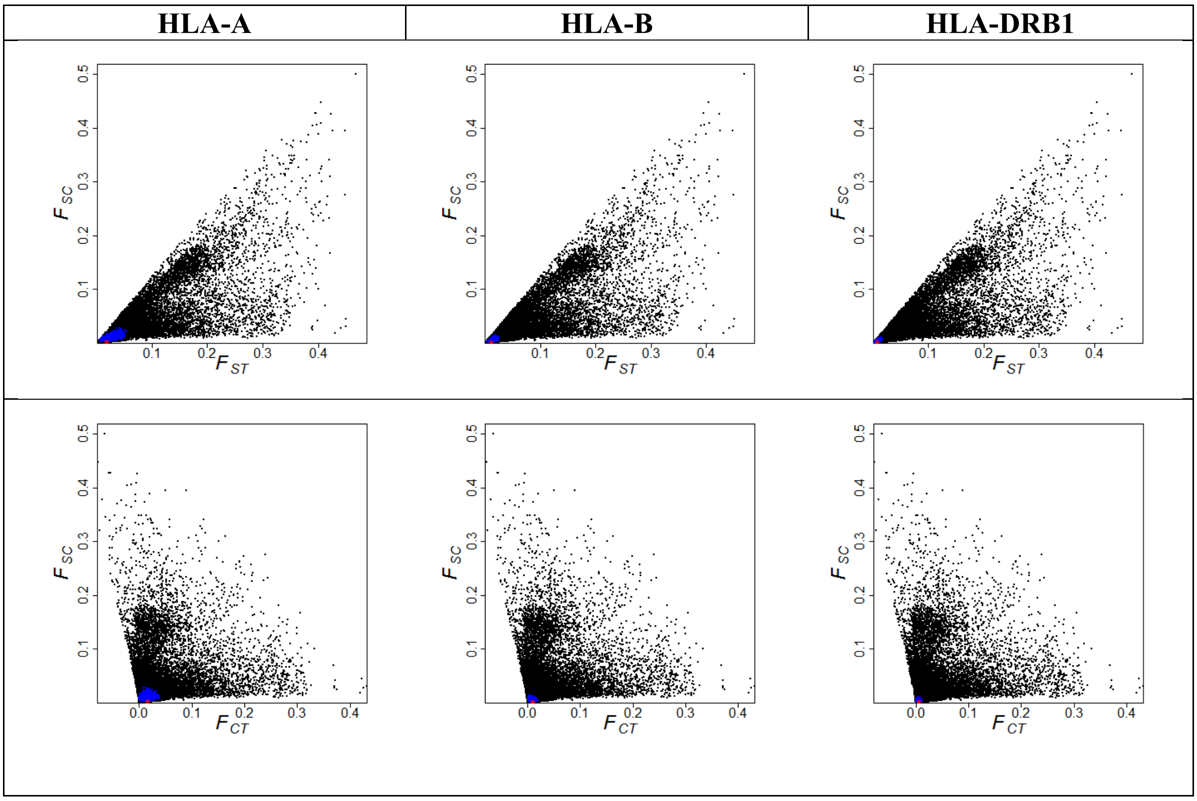
HLA-A
HLA-B
HLA-DRB1
F SC
F ST
F CT
0.0
0.1
0.2
0.3
0.4
0.5
