# Supplementary material for: Computer simulation of human leukocyte antigen genes supports two main routes of colonization by human populations in East Asia
Source: BMC Evol Biol. 2015 Nov 4;15:240. doi: 10.1186/s12862-015-0512-0 (PMC4632674; doi:10.1186/s12862-015-0512-0)
Supplement: Additional file 4: Table S4. — Model comparison using the Latitudinal Overdominant Selection model (LOS). (PDF 59 kb) [file 12862_2015_512_MOESM4_ESM.pdf]

Table S4 Model comparison using the Latitudinal Overdominant Selection model (LOS). To simulate latitudinal overdominant selection, the value of the two selection coefficients against homozygotes are drawn from the same prior distribution (Table 1) and attributed to the northern and southern deme, respectively. Then the strength of the selection coefficient is computed from a linear regression between those two values, regarding the latitude of the corresponding deme. Proportions of simulations (%) under each of the three models among 600 and 1200 best simulations retained from 120,000 simulations (40,000 for each model under LOS) are listed

Number of retained simulations	Locus	Southern-origin model	Pincer model	Overlapping model
600	A	0.5	43.2	56.3
	B	0	30.0	70.0
	DRB1	0	36.7	63.3
1200	A	0.6	46.7	52.7
	B	0	39.0	61.0
	DRB1	0	35.3	64.7
