# Supplementary material for: Computer simulation of human leukocyte antigen genes supports two main routes of colonization by human populations in East Asia
Source: BMC Evol Biol. 2015 Nov 4;15:240. doi: 10.1186/s12862-015-0512-0 (PMC4632674; doi:10.1186/s12862-015-0512-0)
Supplement: Additional file 5: Table S5. — Model comparison using the Negative Frequency-Dependent Selection model (NFDS). (PDF 75 kb) [file 12862_2015_512_MOESM5_ESM.pdf]

**Table S5 Model comparison using the Negative Frequency-Dependent Selection model (NFDS).** To simulate frequency-dependent selection the probability of keeping a new allele for each new individual is computed as $p = 1\text{-}f(a_i)*s$ where $f(a_i)$ is the current frequency of allele $a_i$ in the deme and $s$ is the selection coefficient against frequent alleles. Proportions of simulations (%) under each of the three models among 150 and 300 best simulations retained from 30,000 simulations (10,000 for each model under NFDS) are listed

| **Number of retained simulations** | **Locus** | **Southern-origin model** | **Pincer model** | **Overlapping model** |
|---|---|---|---|---|
| 150 | A | 12.0 | 41.3 | 46.7 |
| | B | 0 | 14.7 | 85.3 |
| | DRB1 | 0 | 38.7 | 61.3 |
| 300 | A | 28.0 | 34.3 | 37.7 |
| | B | 0 | 23.7 | 76.3 |
| | DRB1 | 0.7 | 44.3 | 55.0 |
