# Supplementary material for: Computer simulation of human leukocyte antigen genes supports two main routes of colonization by human populations in East Asia
Source: BMC Evol Biol. 2015 Nov 4;15:240. doi: 10.1186/s12862-015-0512-0 (PMC4632674; doi:10.1186/s12862-015-0512-0)
Supplement: Additional file 6: Table S6. — RMSEP charts of the parameters under the Overlapping model. (PDF 158 kb) [file 12862_2015_512_MOESM6_ESM.pdf]

Table S6 RMSEP charts of the parameters under the Overlapping model

Parameter	RMSEP chart	Parameter	RMSEP chart
**Lineage number *A***	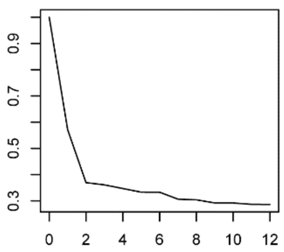	**Selection coefficient *s***	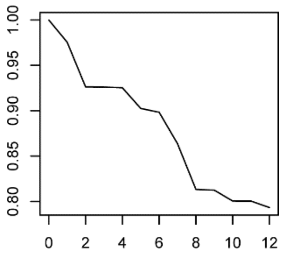
**Genetic exchange time *T* between NEAs and SEAs**	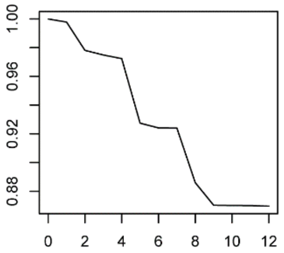		
**Demographic density *N* (NEA)**	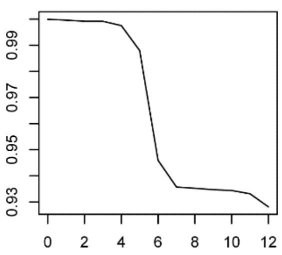	**Migration rate *m* (NEA)**	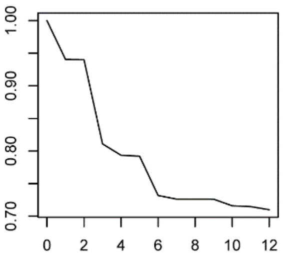
***Nm* (NEA)**	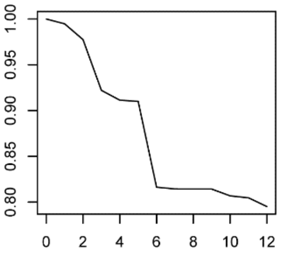	**Growth rate *r* (NEA)**	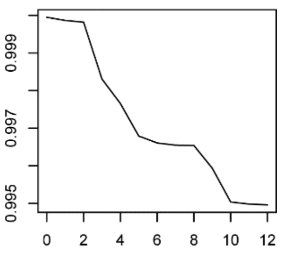
**Demographic density *N* (SEA)**	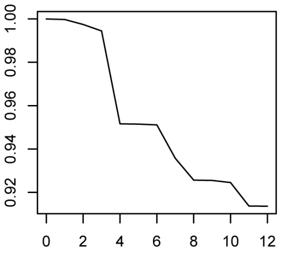	**Migration rate *m* (SEA)**	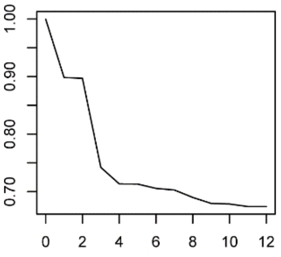

Parameter	RMSEP chart	Parameter	RMSEP chart
Nm<br>**(SEA)**	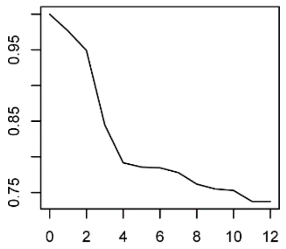	**Growth rate *r***<br>**(SEA)**	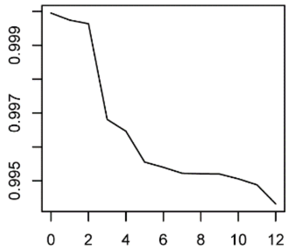
**Demographic density *N***<br>**(41-45°N area)**	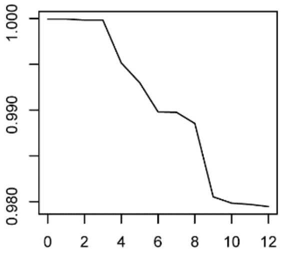	**Migration rate *m***<br>**(41-45°N area)**	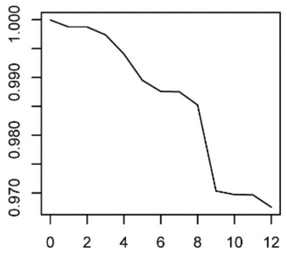
Nm<br>**(41-45°N area)**	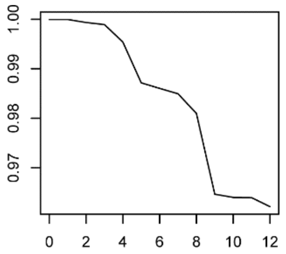	**Growth rate *r***<br>**(41-45°N area)**	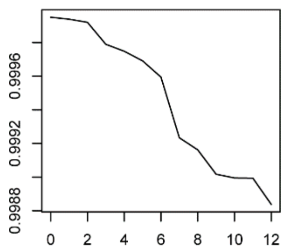

In each chart, X axis represents PLS numbers Y axis shows influence of the parameters on the variances of statistics.
