# Supplementary material for: Computer simulation of human leukocyte antigen genes supports two main routes of colonization by human populations in East Asia
Source: BMC Evol Biol. 2015 Nov 4;15:240. doi: 10.1186/s12862-015-0512-0 (PMC4632674; doi:10.1186/s12862-015-0512-0)
Supplement: Additional file 7: Table S7. — Results of Kolmogorov-Smirnov test against a uniform distribution. (PDF 115 kb) [file 12862_2015_512_MOESM7_ESM.pdf]

Table S7 Results of Kolmogorov-Smirnov test against a uniform distribution. The pseudo-observation density is represented graphically under the Overlapping model using 10-quantiles

**Para-meter**	**Significance**	**Distribution of quantiles**	**Para-meter**	**Significance**	**Distribution of quantiles**
A	Significant deviation from uniformity with too many true values falling in the middle of the posterior distribution ($p < 0.001$)	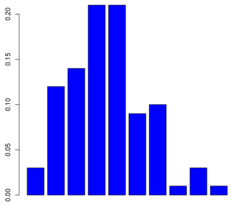	***S***	-	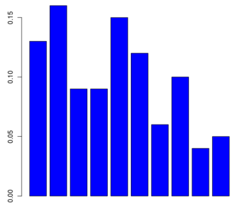
T	-	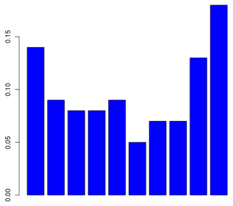			
Nm **(NEA)**	-	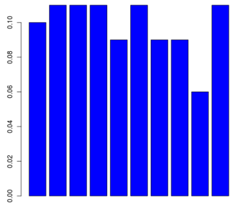	***r*** **(NEA)**	-	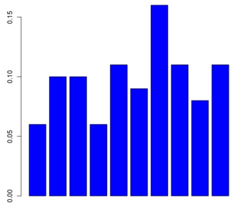
Nm **(SEA)**	-	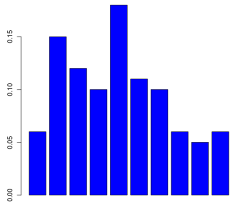	***r*** **(SEA)**	-	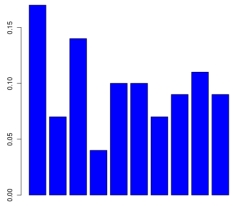
Nm **(41-45°N area)**	-	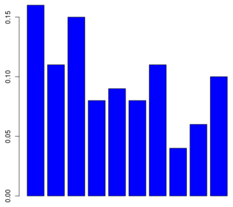	***r*** **(41-45°N area)**	-	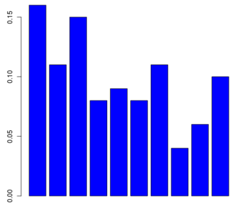
